# Comparison of anadromous and landlocked Atlantic salmon genomes reveals signatures of parallel and relaxed selection across the Northern Hemisphere

**DOI:** 10.1111/eva.13129

**Published:** 2020-09-23

**Authors:** Erik Kjærner‐Semb, Rolf B. Edvardsen, Fernando Ayllon, Petra Vogelsang, Tomasz Furmanek, Carl Johan Rubin, Alexey E. Veselov, Tom Ole Nilsen, Stephen D. McCormick, Craig R. Primmer, Anna Wargelius

**Affiliations:** ^1^ Institute of Marine Research Bergen Norway; ^2^ Uppsala University Uppsala Sweden; ^3^ Institute of Biology of the Karelian Research Centre Petrozavodsk Russia; ^4^ Department of Biological Sciences University of Bergen Bergen Norway; ^5^ Conte Anadromous Fish Research Laboratory U.S. Geological Survey, Leetown Science Center Turners Falls MA USA; ^6^ Organismal and Evolutionary Biology Research Program Faculty of Biological and Environmental Sciences University of Helsinki Helsinki Finland; ^7^ Institute of Biotechnology University of Helsinki Helsinki Finland

**Keywords:** disease resistance, freshwater resident, GWAS, pool sequencing, *Salmo salar*, seawater adaptation, selective sweeps, smoltification

## Abstract

Most Atlantic salmon (*Salmo salar* L.) populations follow an anadromous life cycle, spending early life in freshwater, migrating to the sea for feeding, and returning to rivers to spawn. At the end of the last ice age ~10,000 years ago, several populations of Atlantic salmon became landlocked. Comparing their genomes to their anadromous counterparts can help identify genetic variation related to either freshwater residency or anadromy. The objective of this study was to identify consistently divergent loci between anadromous and landlocked Atlantic salmon strains throughout their geographical distribution, with the long‐term aim of identifying traits relevant for salmon aquaculture, including fresh and seawater growth, omega‐3 metabolism, smoltification, and disease resistance. We used a Pool‐seq approach (*n* = 10–40 individuals per population) to sequence the genomes of twelve anadromous and six landlocked Atlantic salmon populations covering a large part of the Northern Hemisphere and conducted a genomewide association study to identify genomic regions having been under different selection pressure in landlocked and anadromous strains. A total of 28 genomic regions were identified and included *cadm1* on Chr 13 and *ppargc1a* on Chr 18. Seven of the regions additionally displayed consistently reduced heterozygosity in fish obtained from landlocked populations, including the genes *gpr132*, *cdca4,* and *sertad2* on Chr 15. We also found 16 regions, including *igf1* on Chr 17, which consistently display reduced heterozygosity in the anadromous populations compared to the freshwater populations, indicating relaxed selection on traits associated with anadromy in landlocked salmon. In conclusion, we have identified 37 regions which may harbor genetic variation relevant for improving fish welfare and quality in the salmon farming industry and for understanding life‐history traits in fish.

## 1 INTRODUCTION

One of the most extreme adaptations in Atlantic salmon (*Salmo salar*) occurred during land rise following the most recent ice age ~10,000 years ago, when numerous salmon strains became landlocked throughout the geographical distribution in the Northern Hemisphere (Hutchings et al., 2019; Tonteri et al., 2005). Since the end of the ice age, landlocked salmon populations have adapted to a life in freshwater, losing selection pressures associated with seawater, marine diets, and seaborne pathogens. It is likely that different landlocked populations of salmon have been exposed to similar selection pressures and relaxed selection on seawater traits and gone through similar genetic adaptation, sometimes independently of each other. Such populations present a unique opportunity to identify genomic regions under selection for different important traits, as successfully demonstrated in salmon for the age at maturity (Ayllon et al., 2015; Barson et al., 2015) and on genes associated with disease resistance (Kjaerner‐Semb et al., 2016; Zueva et al., 2018).

Previous studies on landlocked salmon populations have found that many of the phenotypic transitions associated with preparatory changes for a life in seawater differ from their anadromous counterparts in immunology (Ronneseth et al., 2005), and morphology and hypo‐osmoregulatory capacity (McCormick et al., 2019; Nilsen et al., 2007, 2008). We hypothesize that developmental traits associated with marine life in ancestral anadromous populations have been lost or suppressed in landlocked salmon due to relaxed selection on seawater traits while advantageous traits have been positively selected. Comparisons between landlocked and anadromous salmon may therefore provide an excellent model for identifying genetic mechanisms underlying evolution of important phenotypic traits during seawater adaptation such as smoltification, resistance to seaborne diseases, and omega‐3 synthesis.

Farming of Atlantic salmon is a growing industry; however, sustainability issues such as seaborne diseases associated with sea‐cage rearing are currently limiting further growth of the industry (Taranger et al., 2015). In the recent past, the industry has also reported an increasing incidence of welfare problems associated with production of fast‐growing, large smolts in modern industrial facilities including osmoregulatory problems, disease, poor growth, and precocious maturity. Domestication of salmon may have affected important traits associated with seawater adaptation such as osmoregulation, disease resistance, growth, reproduction, and behavior (Glover et al., 2017). Currently, we do not understand the genetics behind key traits for aquaculture, for example, smoltification, which is a key step in the transition into seawater and if not properly controlled by farmers will result in reduced growth and high mortality in the sea phase. Hence, there is an increasing demand to explain the genetic basis of traits relevant to current aquaculture production, which may support selective breeding programs aiming to increase the welfare and survival of farmed fish.

Here, we have sequenced and compared genomes of anadromous and landlocked salmon populations throughout their geographical distribution. We found several genes and genomic regions where all the assayed landlocked populations show signs of parallel selection. We also identified genes potentially important during the marine phase by screening for regions showing consistently relaxed purifying selection in landlocked compared to anadromous salmon.

## 2 MATERIALS AND METHODS

### 2.1 Sample collection and DNA extraction

**FIGURE 1:**
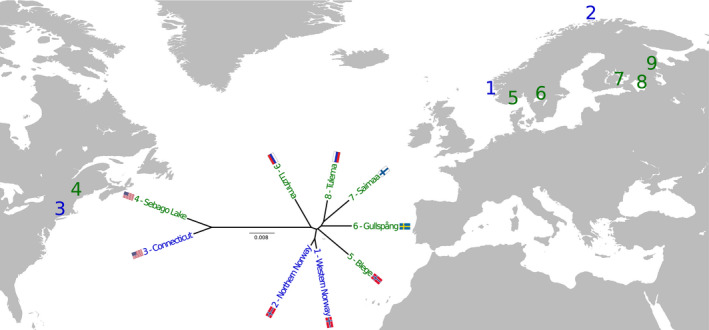
Geographical overview of salmon populations. Sequenced genomes of Atlantic salmon from six landlocked populations (green) and 12 anadromous populations (combined into three groups, blue) were analyzed in this study and are indicated by numbers on a map of the Northern Hemisphere. Genetic distances between the populations are illustrated as a phylogenetic tree based on pairwise calculations of fixation index (*F*_ST_), where the scale bar indicates *F*_ST_. Organization of subpopulations is illustrated in Figure S1, and a more detailed description of the populations is given in Table 1 and Table S1

All tissue samples have been obtained from scientific sampling or from professional or recreational fishers, except the landlocked fish from Gullspång and Blege, which were reared in freshwater in our hatchery facility (Matredal, Norway) for one (Blege) and two generations (Gullspång) under conditions similar to standard commercial fish farming, and are therefore exempt from the Norwegian Regulation on Animal Experimentation (NARA). Rearing and sampling of salmon from Connecticut River and Sebago Lake have been described previously (McCormick et al., 2019) and were in accordance with U.S. Geological Survey (USGS) institutional guidelines and protocol LSC‐9096 that was approved by the USGS Leetown Science Center Institutional Animal Care and Use Committee. Genomic DNA was extracted from scales or fins collected from fish representing all the populations included in this study using one of several methods including Qiagen DNeasy Blood and Tissue or Mini Kits (Qiagen), or a salt‐based extraction protocol as performed by Tonteri et al. (2005). Populations are shown on a map of the Northern Hemisphere in Figure 1 and are listed in Table 1, with a more detailed description in Table S1. A schematic overview of the organization of populations and analyses is presented in Figure S1.

**TABLE 1:** Anadromous and landlocked populations analyzed by pooled whole‐genome sequencing

Population	Short name	Country	Anadromy	Number of pools of 10 individuals	Depth of coverage for SNPs
Western Norway	WN	Norway	Anadromous	24	244
Northern Norway	NN	Norway	Anadromous	15	134
Connecticut River	CON	USA	Anadromous	2	32
Sebago Lake	SEB	USA	Landlocked	2	30
Gullspång	GUL	Sweden	Landlocked	2	23
Blege	BLE	Norway	Landlocked	2	25
Luzhma	LUZ	Russia	Landlocked	1	18
Saimaa	SAI	Finland	Landlocked	1	17
Tulema	TUL	Russia	Landlocked	1	17

Note:

WN contains four pools from each of 6 populations from Western Norway, and NN contains 3 pools from each of five populations from Northern Norway. The average depth of coverage for SNPs is given as peak values (the depth value that was most prevalent in each sample), as visualized in Figure S2. A more detailed description of all populations used in this study is presented in Table S1 and illustrated in Figure S1.

### 2.2 Pooled genome sequencing

DNA purity was assayed using Nanodrop (Thermo Fisher), and fluorometric quantification with Qubit (Thermo Fisher) was used to measure DNA concentrations of each sample. DNA pools were made by pooling equal amounts of genomic DNA from 10 individuals from the same population (Rubin et al., 2010). One to four pools were made for each population, and DNA integrity was inspected by gel electrophoresis. Paired‐end libraries were made for each pool using Genomic DNA Sample Preparation Kit (Illumina), Regular TruSeq Adapter Ligation Kit (Illumina), or with TruSeq PCR‐free kit (Illumina), and sequenced as 100–150‐bp paired‐end reads using the Illumina HiSeq platform versions HiSeq 2000, HiSeq 2500 and HiSeq X at the Norwegian Sequencing Center (https://www.sequencing.uio.no). The sequenced pools of anadromous salmon from Norway have been used in previous studies (Ayllon et al., 2015; Kjaerner‐Semb et al., 2016) and include only males, while the rest of the populations either contain separate pools of males and females, or pools where males and females have been mixed. Atlantic salmon lack typical sex chromosomes, but instead contains the sex‐determining sdy‐locus, which is the only difference between males and females (Yano et al., 2013). Therefore, it is unlikely that any other regions are highly different between male and female salmon. All sequence data used in this study are available on SRA (BioProject ID: PRJNA627844), with accession numbers of all sequenced pools listed in Table S1.

### 2.3 Processing of sequence data and SNP calling

To minimize batch effects from the use of different versions of the Illumina HiSeq sequencing platform, stringent filtering steps were applied to the data. Quality analysis of the sequence data, including screening for degenerated adapter sequences, was done using FastQC (https://www.bioinformatics.babraham.ac.uk/projects/fastqc/). Read pairs were filtered using Cutadapt (v. 1.18) (Marcel, 2011) with the following specifications: The first and last two bases of each read were removed (using the parameters ‐u 2 ‐u −2 ‐U 2 ‐U −2), and low‐quality bases were trimmed from the 3’ end of each read by setting the option −q to 25. Minimum overlap between adapter and read sequences was set to 15 bp using the ‐O option, and reads containing adapters and reads shorter than 75 bp were discarded (‐‐discard and ‐m options, respectively). Filtered reads were mapped to the Atlantic salmon reference genome (v. ICSASG_v2) (Lien et al., 2016) using Bowtie2 (v. 2.3.4.3) (Langmead & Salzberg, 2012) with default parameters. The mapped reads were further processed with Samtools (v. 1.9) (Li et al., 2009) for duplicate removal, quality filtering, and SNP calling as follows: First, the alignment files were converted to BAM format with ‘samtools view’ using the ‐b option, followed by sorting by read names using ‘samtools sort’ with the ‐n option. Read mate information was updated using ‘samtools fixmate’ with the ‐m option, followed by coordinate sorting with ‘samtools sort’, before marking duplicated reads with ‘samtools markdup’. Finally, reads were filtered with ‘samtools view’ with the −q option set to 20 to remove reads with ambiguous mapping and setting the ‐F option to 1,024 to remove duplicated reads. SNPs were called for each population using ‘samtools mpileup’ with the ‐a and ‐B options, and minimum base and mapping quality thresholds (‐Q and ‐q options, respectively) of 20. The resulting mpileup file was further converted to sync format (as used in PoPoolation2) (Kofler et al., 2011) and filtered (using custom scripts) by retaining only SNPs having global minor allele counts of at least 2 and exactly two alleles. Pools belonging to the same population were merged by summing the allele counts of the pools. Subsequently, the anadromous populations from Western Norway (*n* = 6) and Northern Norway (*n* = 5) were grouped as two populations by summing the allele counts for the populations contained in each of the two groups (since they had been sequenced in the same way, with similar depths of coverage), resulting in a total of 9 populations (illustrated in Figure S1 and listed in Table 1, with more details shown in Table S1). Finally, each SNP was required to have a minimum depth of coverage of 5 in each of the 9 populations (i.e. SNPs with coverage less than 5 in any populations were discarded from the entire dataset), and SNPs in unplaced scaffolds or in mitochondrial DNA were discarded. The SNPs were annotated and divided into functional categories with SnpEff (v. 4.2) (Cingolani et al., 2012) using the Atlantic salmon reference genome annotation.

### 2.4 Phylogenetic analysis of sequenced populations

*F*_ST_ was calculated (with a custom Python script, Script S1) for all pairwise comparisons which included all the identified SNPs, using the formula $F_{\text{ST}}=\frac{\bar{p}(1‐\bar{p})‐\bar{p(1‐p)}}{\bar{p}(1‐\bar{p})}$ presented in (Nei, 1977), for each SNP, where *p* represents the allele frequency of the reference allele for each of the two populations in each pairwise comparison. *F*_ST_ values of all SNPs were averaged for each pairwise comparison to make a distance matrix. The distance matrix was used to generate a neighbor‐joining tree using Neighbor from the Phylip package (v. 3.696) (Felsenstein, 2005) and the tree was visualized in Geneious (v. 10.2.4) (Kearse et al., 2012).

### 2.5 Identifying differentiated SNPs and selective sweeps

Identification of SNPs that were differentiated between anadromous and landlocked populations was done by calculating the difference in allele frequency between the two groups (dAF) (Carneiro et al., 2014) using the formula $\text{dAF}=\left|p_{L}‐p_{A}\right|$ for each SNP, where *p*_L_ and *p*_A_ are the average reference allele frequencies of the landlocked (*n* = 6) and anadromous (*n* = 3) populations, respectively. Our aim was to uncover differentiated genomic regions, indicating selective sweeps, so we performed a genomewide screen for regions containing several highly differentiated SNPs. Selective sweeps were predicted in 100 kb sliding genomic windows with 50 kb step size, only considering windows having at least 10 SNPs with a minimum dAF of 60%. Each window was then extended 50 kb to each side, and overlapping windows were merged. Regions passing these criteria were considered as putative selective sweeps.

### 2.6 Pooled heterozygosity

In order to ensure high quality of the data, SNPs with inconsistent depths of coverage were removed from the initial set of SNPs by using strict filtering with the requirement that the depth of coverage for each SNP had to be within one standard deviation of the peak depth for each population (Figure S2). If a SNP had depth of coverage outside this threshold in any population, it was discarded from the entire dataset. Heterozygosity was calculated in 50 kb sliding genomic windows with a step size of 1 bp. Using 1 bp step size provides a much higher genomic resolution as it includes all possible genomic windows, and is explored in more detail in Qanbari et al. (2012). Windows having low numbers of polymorphic loci are more susceptible to spurious fixation signals and uncertain heterozygosity values, so to increase the confidence of the analysis, windows having fewer than 10 SNPs were discarded (Qanbari et al., 2012; Rubin et al., 2010). For each population, the pooled heterozygosity of a window (Hp) was calculated with the formula $\text{Hp}=\frac{1}{n}\sum_{i=1}^{n}2p_{i}\left(1‐p_{i}\right)$, where *p_i_* is the allele frequency of the global major allele for the *i*‐th SNP in a given window containing *n* SNPs. This is similar to what has been done in Qanbari et al. (2012) and Rubin et al. (2010), except that we calculate the heterozygosity for each SNP and take the average for each window.

To control for background levels of genetic diversity differences between populations caused by genetic drift, Hp values for each population were normalized by conversion to *Z*‐scores (ZHp) using the formula $\text{ZHp}=\frac{\text{Hp}‐\mu_{\text{Hp}}}{\sigma_{\text{Hp}}}$ for each genomic window in a population, where *µ*_Hp_ is the mean and *σ*_Hp_ is the standard deviation of all the Hp values in a given population, resulting in a distribution of ZHp where *µ*_ZHp_ = 0 and *σ*_ZHp_ = 1 for each population (Rubin et al., 2010). The difference in ZHp values (dZHp) between landlocked and anadromous populations was determined with the formula $\text{dZHp}=\bar{{\text{ZHp}}_{A}}‐\bar{{\text{ZHp}}_{L}}$, where ZHp_A_ and ZHp_L_ are the ZHp values of a given genomic window from anadromous and landlocked populations, respectively. Since outlier values can have a strong influence on the average, and since we were interested in regions showing consistent signs of differentiated heterozygosity, windows were considered to be consistently differentiated if they passed the following criteria: For a given window, each of the populations in one group should have ZHp above their respective population averages, while each of the populations in the other group should have ZHp below their respective population averages. Since the average ZHp of all windows in any given population is 0 because of the conversion of Hp values to *Z*‐scores, this means that all populations in the first group should have ZHp values > 0, and all populations in the other group should have ZHp values < 0, for a given window to be considered differentiated. Overlapping windows with differentiated ZHp values were merged using the ‘intersect’ tool from Bedtools, into regions with reduced heterozygosity in either anadromous or landlocked salmon.

Differentiated regions under selection are often characterized by a reduction in heterozygosity in the populations experiencing the selective pressure and are an indication of adaptive divergence (Kjaerner‐Semb et al., 2016; Smith & Haigh, 1974). To identify regions undergoing adaptive divergence, regions with consistently reduced ZHp were compared with regions containing differentiated SNPs by intersecting the lists of regions with the ‘intersect’ tool from Bedtools.

One of our main aims was to identify regions with consistently reduced heterozygosity in anadromous populations. Because our dataset only included three anadromous populations, we extended the number of populations by including additional populations from a study performed by Zueva et al. (2018), in which the authors screened the salmon genome for signatures of parasite‐driven selection in north European salmon using a 220K SNP array. The dataset contains several anadromous salmon populations from Barents Sea (*n* = 10) and White Sea (*n* = 22) and landlocked populations from the Russian lakes Ladoga (*n* = 6) and Onega (*n* = 5). Each population is represented by allele frequencies of DNA pools of >22 individuals per population obtained from a SNP array comprising 197,431 SNP markers. To analyze the data, allele frequencies were averaged over the populations from each of the four groups (Barents Sea, White Sea, Ladoga, and Onega) and heterozygosity was analyzed similarly to what was done for the sequenced populations in the present study. Briefly, Hp was calculated in 50 kb sliding genomic windows with 1 bp step size followed by conversion to *Z*‐scores. Genomic windows where max(ZHp_A_) < 0 and min(ZHp_L_) > 0, where ZHp_A_ and ZHp_L_ are the ZHp values of a given genomic window from anadromous and landlocked populations, respectively, were considered as having reduced heterozygosity in the anadromous salmon. Regions with consistent reduction in heterozygosity in anadromous salmon were defined as regions with reduced ZHp in anadromous salmon in our sequence‐based data that overlapped with windows showing reduced ZHp in anadromous salmon from the dataset presented in Zueva et al. (2018). The same approach was used to identify regions with consistently reduced ZHp in landlocked populations. Genomic windows passing the criteria max(ZHp_L_) < 0 and min(ZHp_A_) > 0, where ZHp_L_ and ZHp_A_ are the ZHp values of a given genomic window from landlocked and anadromous populations, respectively, were considered as having consistently reduced heterozygosity in the landlocked salmon.

### 2.7 Genotyping individual fish

To obtain individual‐specific genotype distributions and to investigate more anadromous and landlocked populations at the genome regions of interest, Custom TaqMan SNP Genotyping Assays (cat. no 4332077, Thermo Fisher) were designed for the SNPs Chr13: 66061636 and Chr15: 41215721, see Results (primers and probes are listed in Table S2). From each of the populations listed in Table S1, 10–61 individuals were genotyped for both SNPs. The genotyping assays were run on QuantStudio 5 (Thermo Fisher).

### 2.8 Gene annotation and tissue‐specific gene expression analysis

The Atlantic salmon reference gene model GFF file (v. ICSASG_v2) (Lien et al., 2016) was used to identify genes in genomic regions of interest by overlapping the GFF file with BED files containing selected regions using the ‘intersect’ tool from the Bedtools package (v. 2.26.0) (Quinlan & Hall, 2010). Genes were annotated by performing alignment searches using BLASTP (Altschul et al., 1997) with the amino acid sequences from the reference gene models against the Swiss‐Prot database (v. 2015.08.10). Tissue‐specific expression profiles of genes in these genomic regions of interest were examined using RNA‐Seq data from various salmon tissues obtained from SRA (BioProject ID: PRJNA72713). Briefly, sequence reads were mapped to the gene models using Bowtie2, and read counts were summed for each gene ID and normalized by total mapped read counts. Heatmaps were made by first discarding genes that had normalized read counts <50 in all assayed tissues, before using J‐Express (v. 2012) (Dysvik & Jonassen, 2001) to generate heatmaps using high‐level mean and variance normalization, with complete linkage clustering and Euclidean distance measure. Gene expression in gills of salmon exposed to saltwater for 24 hr was examined using RNA‐Seq data obtained from Array Express (accession number E‐MTAB‐8276), described previously in Iversen et al. (2020). Sequence reads were filtered using Cutadapt with parameters ‐q 20 ‐O 8 and ‐m 40 and mapped to the salmon gene models with Bowtie2 using default settings. DESeq2 (Love et al., 2014) was used to identify differentially expressed genes between fish exposed to saltwater (*n* = 84) and controls (*n* = 83) divided into six different sampling points (the fish were approximately 7 months of age at experiment start). Read counts were summed for each gene ID and normalized by total mapped read counts.

### 2.9 Determination of missense SNP ancestral state

Ancestral state of a missense SNP in the candidate gene *cadm1* (see Results) was determined by aligning the Cadm1 amino acid reference sequence (accession: XP_013992853) against the refseq_protein database using BLASTP (https://blast.ncbi.nlm.nih.gov), only including matches to teleost fishes (taxid: 32443).

## 3 RESULTS AND DISCUSSION

Landlocked salmon have been isolated in freshwater lakes for millennia, where they have been shaped by subsequent evolution as they adapted to a life without oceanic migration. Traces of the underlying evolutionary forces they have been subjected to including those they are no longer influenced by can be revealed by genome sequencing. By pooled whole‐genome sequencing, we have compared the genomes of 6 landlocked and 12 anadromous salmon populations from a wide geographical range across the Northern Hemisphere (Figure 1, Figure S1, Table 1) and uncovered genes and genomic regions with signs of selection and adaptation in response to life with or without marine migration.

Sequence reads mapped to the Atlantic salmon reference genome were used to identify a total of 43,329,247 single nucleotide polymorphisms (SNPs) in the genomes of landlocked and anadromous salmon. Atlantic salmon inhabit the entire coast of Norway; however, due to gene flow between neighboring populations, they are quite homogeneous and were therefore divided into two major groups representing the western and northern Norwegian populations (Kjaerner‐Semb et al., 2016; Wennevik et al., 2019). Phylogenetic analysis showed that the genetic relationship between the populations included in this study corresponded with expected geographical distributions and colonization patterns (Bourret et al., 2013), with the greatest differentiation between the western and eastern Atlantic populations (Figure 1). It further showed that despite relatively small geographical separation, the Luzhma and Tulema populations were phylogenetically quite far apart, supported by previous reports showing that these populations likely originate from different postglacial refugia in the Eastern Barents‐White Sea and Baltic Ice Lake, respectively (Bourret et al., 2013; Tonteri et al., 2005).

### 3.1 Genomewide SNP analysis reveals 28 selective sweeps

**FIGURE 2:**
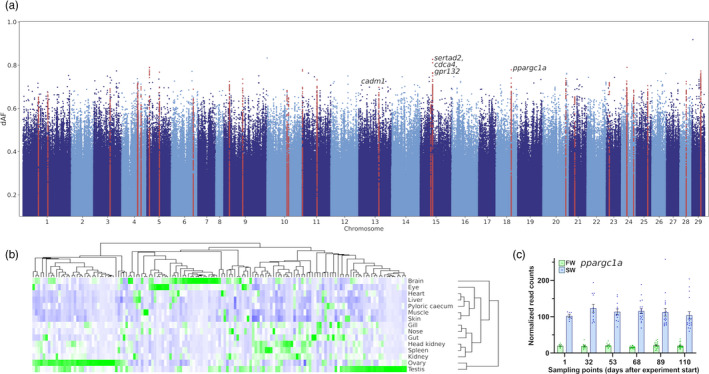
Differentiated genomic regions. (a) Manhattan plot showing SNP allele frequency differences (dAF) between landlocked and anadromous populations of Atlantic salmon in the Northern Hemisphere. The *x*‐axis shows chromosomal positions along the salmon genome, and the *y*‐axis shows the difference in allele frequencies between the two groups. SNPs in selective sweep regions (*n* = 28), identified using a threshold of dAF > 0.6 using 100 kb nonoverlapping genomic windows are marked in red. (b) Heatmap showing tissue distribution of normalized gene expression of genes in the identified selective sweeps. Green = increased expression, blue = reduced expression. A detailed view of the heatmap including gene IDs is shown in Figure S4. (c) Upregulation of *ppargc1a* in gills after 24 hr saltwater exposure. The *y*‐axis shows normalized read counts for *ppargc1a* in salmon gills, and the *x*‐axis shows the sampling points given as number of days since experiment start. Blue indicates salmon challenged with saltwater (SW) for 24 hr and green indicates salmon kept in freshwater (FW). Contrasts between FW and SW were significant at each sampling point (*p*_adj_ < 4.38E‐41)

Identification of differentiated SNPs was based on the difference between average SNP allele frequencies between two groups (dAF). This allowed us to identify parallel selection on genetic variation in multiple landlocked populations, where SNPs present in the ancestral anadromous populations were subjected to strong positive selection for the same allele after the formation of the landlocked populations. We used two different thresholds for reporting differentiated SNPs; dAF > 0.5, which resulted in 15,038 SNPs, and dAF > 0.6, resulting in 2,194 SNPs. Regions harboring at least ten differentiated SNPs (dAF > 0.6) in 100 kb sliding genomic windows were regarded as selective sweeps, and genomewide screening revealed 28 sweeps containing many differentiated SNPs, potentially resulting from different selection pressures in the landlocked and anadromous populations (Figure 2a, Table 2).

**TABLE 2:** Selective sweeps differentiated between anadromous and landlocked populations. Regions harboring ≥ 10 SNPs having dAF values ≥ 0.6 identified in 100 kb nonoverlapping genomic windows

Chromosome	Chromosomal region	Sweep length (bp)	Genes
1	50,450,000–50,700,000	250,000	dntt, hs3st1
1	81,550,000–81,800,000	250,000	rab4a, psmb1‐a, rhou, unknown, phf10, tbp, ccsap, pdcd2, galnt2, act2
3	54,000,000–54,250,000	250,000	csf2rb2, dnal4, unknown, baiap2l2, unknown, nptxr, ms4a12, ms4a12, trim33
4	51,750,000–51,950,000	200,000	shpk, ncor1, unknown, ubi‐p63e, p2rx5, emc6, trpv1, pigl, trpv1
4	52,450,000–52,850,000	400,000	unknown, mrpl22, gemin5, unknown
**4**	**63,300,000–63,500,000**	**200,000**	**arhgef12, tmem136, oaf, unknown, pde9a, slc37a2, pou2f3, unknown, hepacam, ccdc15**
**5**	**8,550,000–8,800,000**	**250,000**	**kif3a, sh3rf1**
5	41,350,000–41,600,000	250,000	il1rapl2
6	72,050,000–72,300,000	250,000	dio3, unknown, hsp90a.1, hsp90a.1, wdr20, mok, slamf6, ppp2r5d, slamf6
9	18,500,000–18,800,000	300,000	mycl1b, unknown, mfsd2ab, marcksl1, nt5c1a
**9^†^**	**62,150,000–62,400,000**	**250,000**	
10	66,100,000–66,300,000	200,000	unknown, unknown, pik3c2a, nucb2, trim16, tdg, api5, samm50a, tdg, hsd17b12a
**10**	**71,250,000–71,450,000**	**200,000**	**glg1, fbln7, tf2−9, afg3l1, cacna2d4, lrtm2**
11	700,000–900,000	200,000	unknown, alk
11	48,450,000–48,850,000	400,000	nrg1, ppp2cb, zbtb43, pat, chd1, pde4d
13	65,950,000–66,200,000	250,000	cadm1
15	34,950,000–35,200,000	250,000	ehd3, galnt14, angel2, vash2, flvcr1, spata45, nsl1, atf3, batf3, tatdn3
**15**	**41,000,000–41,350,000**	**350,000**	**sertad2, ism2, sptlc2, sel1l, znf706, ahsa1, vipas39, cdca4, snw1, sel1l, plb1, gpr132**
18	49,600,000–50,100,000	500,000	frem1, unknown, rap1gds1, ppargc1a, htr3a, bmp4, tspan5, znf135, znf180, zmym1, znf135, ankhd1, znf596, dhx15, unknown, ccdc149b
20	75,350,000–75,600,000	250,000	wdr49, pdcd10
21	18,050,000–18,300,000	250,000	
23	9,950,000–10,200,000	250,000	lurap1, ttc4, rln3, unknown, unknown, sgip1, pars2, unknown
**24^†^**	**17,450,000–17,800,000**	**350,000**	**unknown, unknown, mlxip, mlxip, rsrc2, zcchc8, clip1** **, hip1r, b3gnt7, setd8, pitpnm2, bcl7a, rilpl2, unknown, cdk2ap1, wdr66**
24	18,400,000–18,650,000	250,000	pitpnb, mn1
24	40,000,000–40,250,000	250,000	ubqln1, frmd3, idnk, gkap1, fbxl2, rasef, tle1, tle4
25	37,700,000–37,950,000	250,000	il1rapl1b, gspb, nr0b1, cxorf21
28	21,050,000–21,300,000	250,000	camkmt, hpse2, fam178a
**29**	**29,250,000–29,650,000**	**400,000**	**zfhx4, unknown, rnf12‐b, ezh2, pdia4**

Note:

Gene symbols of genes from the reference annotation are shown as obtained from the annotation against Swiss‐Prot, where genes lacking a gene symbol are indicated by “unknown”. Sweeps overlapping regions with reduced ZHp in landlocked salmon (*n* = 7) are presented in bold. Sweeps overlapping regions with reduced ZHp in both our data and the data from (Zueva et al., 2018; *n* = 2) are indicated by †. A detailed description of the genes can be found in File S2.

### 3.2 Pooled heterozygosity—identifying regions under recent directional selection

**FIGURE 3:**
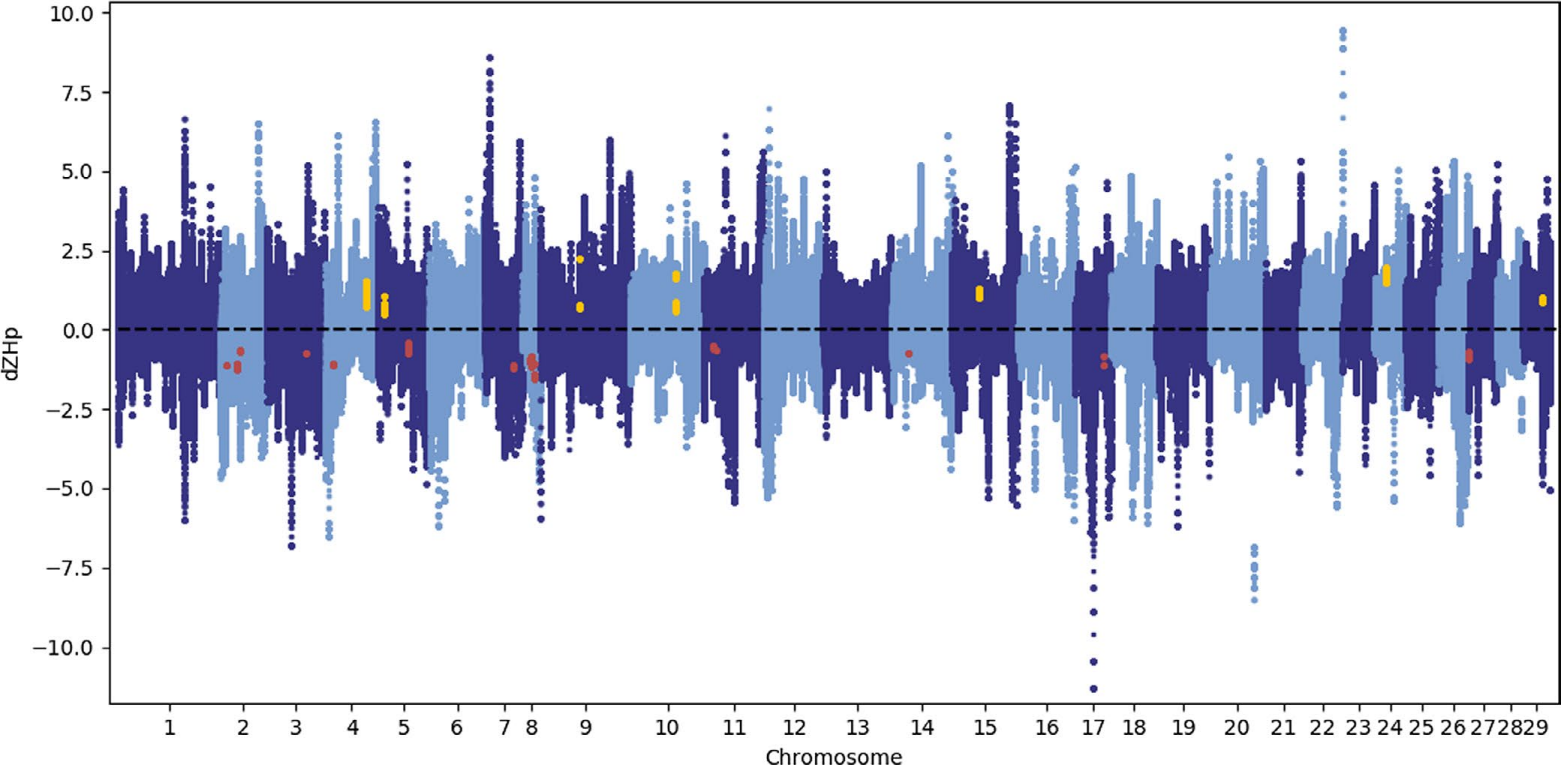
Differences in ZHp between landlocked and anadromous salmon. Manhattan plot showing regions with differentiated ZHp between landlocked and anadromous salmon in 50 kb sliding windows with 1 bp step size along the chromosomes of the Atlantic salmon genome. The *x*‐axis shows the position in the genome, and the *y*‐axis presents the difference in average heterozygosity (dZHp) between the two groups, where regions with low ZHp in landlocked salmon are present above 0 and regions with low ZHp in anadromous salmon are present below 0. Regions with consistently reduced heterozygosity in landlocked salmon that overlapped with any of the 28 selective sweeps are shown as yellow dots, and regions with consistently reduced heterozygosity in anadromous salmon (intersect of our data and data from Zueva et al. (2018)) are shown as red dots. The regions highlighted by yellow and red dots are listed in Table 2 (in bold) and Table 3, respectively

Pooled heterozygosity (Hp) can be used as a measure of the amount of genetic variation present in a given region of the genome of a population, where reduced Hp can indicate recent directional selection (Qanbari et al., 2012; Rubin et al., 2010). The approach used in this study was based on 50 kb sliding windows with 1 bp step size across the entire genome after strict filtering on depth of coverage from the sequencing (Figure S2), and excluding windows having less than 10 SNPs, retaining 10,589,760 (25%) SNPs resulting in a total of 2,161,561,871 genomic windows. To be able to compare the different groups, and to reduce the noise from genetic drift, Hp values were normalized by conversion to *Z*‐scores (ZHp) for each population. Difference in heterozygosity was quantified based on the difference of average ZHp between the two groups in the genomic windows (dZHp) (Figure 3). To identify regions with consistently reduced heterozygosity in one of the two groups, we screened for regions where all populations in one group had lower than average ZHp and all populations in the other group had greater than average ZHp (and vice versa; Figure 3). We believe that this approach provides consistent results, as it is not dependent on any given arbitrary cutoffs, and it avoids bias caused by outlier samples. The analysis of heterozygosity revealed 481 regions showing reduced levels of normalized heterozygosity in the landlocked compared to the anadromous populations, and 485 regions having reduced normalized heterozygosity in anadromous populations compared to the landlocked populations (regions are listed in File S1). Reduced levels of heterozygosity in a region containing differentiated SNPs indicate relatively recent selection and can be used to infer which populations that have experienced the selective forces (Rubin et al., 2010). When the regions with consistently reduced heterozygosity were compared to the 28 selective sweeps found by screening for regions with differentiated SNPs, 7 and 0 of the sweeps overlapped with regions having reduced heterozygosity in landlocked and anadromous salmon, respectively (Figure 3 and Table 2). Interestingly, all the overlapping regions were found in landlocked salmon, indicating that these have experienced parallel selection on the same alleles, and potentially contain genetic variants that are favorable for life in freshwater only.

### 3.3 Regions with consistently relaxed selection in landlocked salmon

Heterozygosity is commonly used as an index of genetic diversity and can also provide indications of purifying selection that keeps genomic regions from accumulating deleterious mutations. If a gene or region becomes less relevant in a population, it is more likely to accumulate mutations that are not purged from the population. This can be used to identify genomic regions that are under purifying selection due to a conserved function of the genes in that region. For example, genes that are vital for survival at sea can be expected to accumulate more mutations in landlocked salmon that no longer require that specific function to be maintained since they no longer migrate to the sea, and therefore experience a reduction in selection pressure. Therefore, we aimed to uncover genomic regions and genes that show increased genetic diversity in landlocked salmon compared to anadromous salmon, potentially leading to discovery of genes associated with seawater‐related traits relevant for aquaculture, such as resistance to seaborne diseases or smoltification.

**FIGURE 4:**
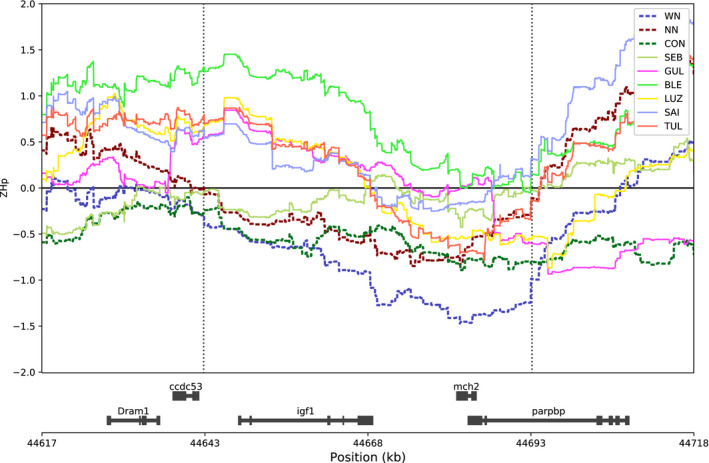
Detailed view of a region showing reduced ZHp in anadromous salmon overlapping *igf1* on Chr 17. Magnification showing a 100 kb region on Chr 17 covering genomic windows with reduced heterozygosity in anadromous salmon. The x‐axis shows the chromosomal positions given in kb and the y‐axis shows the normalized heterozygosity values (ZHp). Each curve presents ZHp values in 50 kb sliding windows (with 1 bp step size) of a population, where the center position of each window is plotted. Anadromous populations are indicated by dotted lines. The horizontal black line indicates the average ZHp of each population (*µ* = 0 after normalization by calculation of *Z*‐scores). Vertical dotted lines indicate the start and end of the region with reduced ZHp in anadromous salmon. Genes from the reference annotation are shown in the bottom. Population codes are explained in Table 1

To narrow down the list of regions showing consistently reduced heterozygosity in anadromous salmon in our data (*n* = 485), and to see whether any of the regions are conserved in other datasets and other populations, we analyzed allele frequency data from Zueva et al. (2018), which includes several additional landlocked populations from the two Russian lakes Ladoga and Onega as well as anadromous populations from the Barents Sea and the White Sea. The populations were grouped into four groups: Ladoga, Onega, Barents Sea, and White Sea, by calculating the average allele frequency for each SNP marker in each group. Heterozygosity was analyzed using the same parameters as for the sequence data in the present study, and the regions with reduced heterozygosity in anadromous salmon that overlapped with our data are reported. In total, 1,217 regions showed reduced heterozygosity in anadromous populations relative to landlocked populations in that dataset, 16 of which overlapped with the regions showing reduced heterozygosity in anadromous populations in our data (shown in Table 3, and indicated by red dots in Figure 3). Since they are conserved in both datasets, these regions are expected to contain potential candidates for genes that are important for the seawater phase. The 16 regions covered 34 genes and, interestingly, included *insulin‐like growth factor 1* (*igf1*) (Figure 4). Igf1 is known to promote the development of salinity tolerance in Atlantic salmon (McCormick, 1996; Sakamoto et al., 1993), and transfer to seawater is associated with increasing plasma levels of Igf1 (McCormick, 2001). Together with growth hormone and cortisol, Igf1 is involved in increasing Na+/K+ ATPase activity in gills in different salmonids to promote seawater tolerance (Bjornsson et al., 1987; Madsen, 1990; McCormick, 1996; Seidelin et al., 1999). Igf1 is also involved in growth regulation of vertebrates including teleost fish (McCormick et al., 1992; Wood et al., 2005), and in farmed Atlantic salmon, SNPs in *igf1* have been associated with overall body weight and fillet weight (Tsai et al., 2014). It is therefore possible to speculate that the gene is conserved in anadromous salmon because of its importance in smoltification and seawater growth, which are processes that have become less relevant for landlocked salmon (Nilsen et al., 2008). Another interesting gene showing consistently reduced heterozygosity in anadromous populations was *TGF‐beta receptor 1* (*tgfbr1*), which is involved in regulation of many different processes in salmonids (Maehr et al., 2012). It has been shown to have a widespread tissue distribution and is highly expressed in the brain and muscle, as well as in immune‐related cells in rainbow trout (Maehr et al., 2012), although in Atlantic salmon the highest expression level was found in ovary (Figure S3). It is also worth noting that of the 16 regions with consistently reduced heterozygosity in anadromous salmon, two of the regions contained paralog regions that were duplicated in the salmonid‐specific whole‐genome duplication (Lien et al., 2016) (Table 3). This indicates that the genes in these regions are under strong purifying selection in anadromous salmon, which has been relaxed in landlocked salmon. The paralog regions overlapped the genes *signal peptidase complex subunit 3* (*spcs3*), *WD repeat domain 17* (*wdr17*), and *ankyrin repeat and SOCS box containing 5* (*asb5*). Their functions in fish are not well characterized; however, *Wdr17* has a function in eyes in mice (Chiang et al., 2020), and there is evidence that spectral sensitivity and eye pigments differ in freshwater and seawater life stages in salmon (Temple et al., 2008). *spcs3* and *asb5* have been assigned to Reactome pathways (https://reactome.org) such as “Viral mRNA Translation,” and “Class I MHC mediated antigen processing and presentation”, respectively, suggesting that these might be related to resistance against seaborne diseases.

**TABLE 3:** Regions with reduced heterozygosity in anadromous salmon. Listing regions showing consistently reduced heterozygosity in anadromous compared to landlocked populations (intersect of our data and the data from Zueva et al. (2018)).

Chromosome	Chromosomal region	Region length (bp)	Genes
2	10,526,090–10,576,406	50,316	clcn1
2	26,241,445–26,298,376	56,931	etv1, dgkb
2	32,506,524–32,558,150	51,626	unknown, macf1, rft2
3	61,880,141–61,931,499	51,358	
4	11,625,820–11,676,588	50,768	spcs3, wdr17, asb5
5	47,905,828–47,964,764	58,936	eif3e, rspo2
7	44,096,893–44,148,649	51,756	ppfia2
8	11,124,829–11,176,404	51,575	asb5, spcs3, wdr17
8	12,454,313–12,553,935	99,622	
8	16,940,959–17,026,461	85,502	unknown, g3bp2, ppm1k, uso1, pkd2, abcg2
11	13,759,622–13,812,520	52,898	cadn
11	17,877,623–17,927,732	50,109	sbf2
14	25,660,660–25,712,400	51,740	tgfbr1
17	44,490,402–44,541,187	50,785	spic, bcl2l13, mybpc3
17	44,642,974–44,693,695	50,721	mch2, igf1, parpbp
27	161,241–236,092	74,851	psmd4, unknown, pip5k1a, znf687

Note:

Gene symbols of genes from the reference annotation are shown as obtained from the annotation against Swiss‐Prot, where genes lacking a gene symbol are indicated by “unknown”. A detailed description of the genes can be found in File S2.

We also identified regions with consistently reduced ZHp in landlocked populations included on the SNP array data presented in (Zueva et al., 2018). In total, 1,274 regions showed consistently reduced ZHp in the landlocked populations (Files S1 and S2), and comparison with ZHp values from the pool‐seq data revealed 63 regions with consistently reduced ZHp in both datasets (Table S3). Further, two of the regions overlapped with selective sweeps identified on Chr 9 and 24 (Table 2). It is worth noting that the relatively low number of marker positions on the SNP array compared to the genomic sequence data restricts the analysis to only regions covered by a sufficiently large number of SNPs on the SNP array.

### 3.4 Tissue‐specific gene expression of genes in selective sweeps

Since genetic variants in the selective sweeps can affect one or more genes inside or outside the identified regions, the genes under selection remain unknown. Inspecting the expression patterns of the genes in the sweeps can offer clues about their function and if they are likely to be involved in a trait under selection. A total of 172 genes were located in the 28 sweep regions, and examining tissue‐specific distribution of gene expression showed that several genes in the sweeps have expression in immune‐related tissues such as spleen, head kidney, and gill, while the majority of the genes were predominantly expressed in brain and gonads (Figures 2b and S4, File S2). These gene expression patterns point to (although not conclusive) selection acting on genes related to traits such as immune response, behavior, and reproduction. We also wanted to investigate if we could observe any tissue‐specific enrichment for genes under selection. Compared to other tissues, gonad and brain express a large number of genes (Lien et al., 2016; Sonawane et al., 2017), which will cause a bias toward genes expressed in those tissues, making it difficult to identify any potential over‐representation of genes under selection in certain tissues. Distribution of tissue‐specific gene expression of a representative set of genes selected by random did not differ from that of genes in the sweeps (Figure S5), indicating that such enrichment is either not present, or the large number of genes in the sweeps that are not under selection masks the enrichment.

### 3.5 Genes in selective sweeps differentially expressed in the gill in response to saltwater

We also screened the sweeps for genes differentially expressed in juvenile fish exposed to saltwater by re‐analysis of a recently published RNA‐Seq dataset (Iversen et al., 2020) from salmon gills. This revealed that 14 of the genes in the sweeps were differentially expressed (*p*_adj_ < .001) in at least one sampling point in fish challenged by saltwater for 24 hr at six sampling points over a 110‐day period (File S3). Strikingly, it further revealed a highly significant upregulation of *pparg coactivator 1 alpha* (*ppargc1a*) at all sampling points (*p*_adj_ < 4.38E‐41, Figure 2c). This gene encodes a transcriptional cofactor located in a sweep on Chr 18 (positions 49,600,000–50,100,000) and is a master regulator of mitochondrial biogenesis and energy expenditure (Fernandez‐Marcos & Auwerx, 2011). Mice lacking this gene show reduced mitochondrial respiratory capacity and an increased expression of lipogenic genes (Leone et al., 2005). Adaptation to seawater is an energy‐demanding process (Hoar, 2008) and salmon smolt show elevated respiratory enzyme activity and mitochondrial proliferation (Maxime et al., 1989), suggesting that *ppargc1a* can be a potential target for selection on salinity tolerance and smoltification.

### 3.6 The most differentiated selective sweep on Chr 15

**FIGURE 5:**
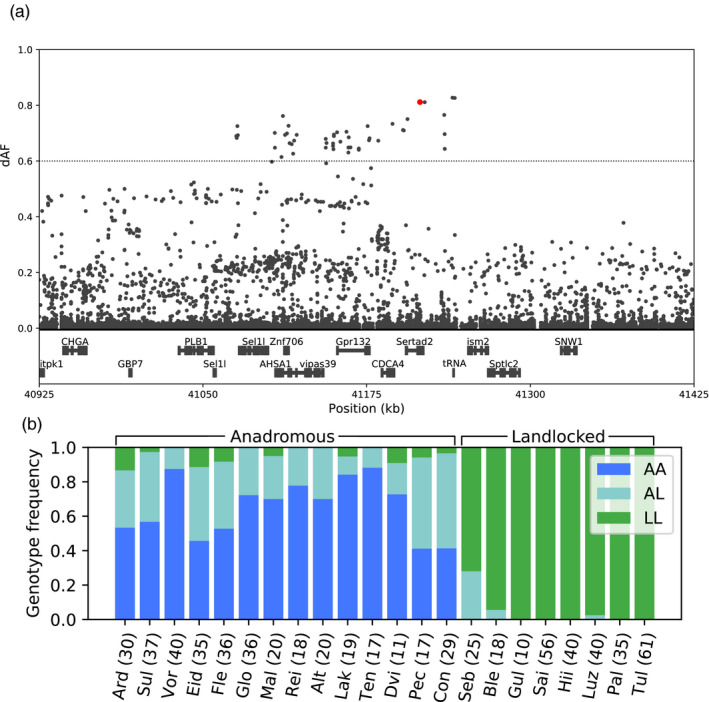
Detailed view of the genomic region on Chr 15 containing SNP alleles near fixation in all landlocked populations analyzed in this study. (a) Magnification showing 500 kb of the selective sweep on Chr 15. SNPs are shown as black dots. The *x*‐axis shows the chromosomal positions given in kb and the *y*‐axis presents the difference in allele frequencies between the two groups (dAF). Genes from the reference annotation are shown in the bottom. (b) Genotype frequencies in different populations based on genotyping of individual fish for a SNP at position 41,215,721 in Chr 15 (indicated by a red dot). AA = homozygous for the anadromous allele, LL = homozygous for the landlocked allele, AL = heterozygous. Population codes are explained in Table S1. The numbers in parentheses show the number of genotyped individuals from each population

The selective sweep with the most differentiated SNPs was found on Chr 15 (positions 41,000,000–41,350,000, Figures 5a and S6), showing reduced heterozygosity in all the sequenced landlocked populations (positions 41,136,048–41,224,312, File S1), indicating that the sweep is under selection in landlocked salmon. Interestingly, in the landlocked Luzhma population, the SNPs in the 5’ half of the sweep to a large extent have the same alleles as the anadromous strains, while the 3’ half of the sweep contains SNPs that are highly differentiated from the anadromous salmon (Figure S6). This suggests that the sweep has been broken up by recombination in the ancestors of the Luzhma population, or during more recent secondary contact, for example, via stocking. Together with the observed reduction in heterozygosity in this region in all the landlocked populations, it seems that the region under selection in the landlocked populations is located at the 3′ side of the sweep, overlapping with *cell division cycle associated 4* (*cdca4*), *SERTA domain‐containing protein 2* (*sertad2*) and a threonine tRNA (GeneID: 106455098), and in close proximity to *G protein‐coupled receptor 132* (*gpr132*). Four SNPs were almost fixed in opposite directions in landlocked and anadromous salmon (dAF > 0.8), even across the Atlantic Ocean. These SNPs were found in the 3′ UTR and 1,160 bp downstream of *sertad2*, and 817 bp upstream and 835 bp downstream of the threonine tRNA. Genotyping a larger number of fish (*n* = 10–61 per population, Table S1) for the SNP in the 3′ UTR of *sertad2* confirmed our observation (Figure 5b). The gene *sertad2* has been shown to modulate adipocyte function, and mice lacking the gene show increased lipolysis (Liew et al., 2013). If the causative variant affects *sertad2* gene regulation differently in landlocked and anadromous salmon, it is possible to imagine a mechanism where reduced expression in landlocked salmon inhibits lipolysis, allowing them to retain their lipid stores, which could be beneficial in a nutrient‐poor environment. The gene *gpr132* encodes a membrane receptor involved in modulation of several biological processes. In mammals, it is highly expressed in macrophages (Bolick et al., 2009; Chen et al., 2017), where it has been shown to facilitate macrophage M2 activation and to have a pro‐inflammatory effect (Chen et al., 2017). In the salmon tissue distribution dataset (Figure S4), we observed higher expression in immune‐related tissues such as spleen and head kidney, suggesting a possible role of this gene in immune defense in salmon. It is possible that different pathogen or parasite exposure in freshwater and seawater has been a driving force for selection on disease resistance (a topic discussed in more detail by Zueva et al. (2018)). Not much is known about *cdca4*, however, the gene encodes a regulator of transcriptional activation involved in cell proliferation (Hayashi et al., 2006) and has been shown to interact with *p53* to promote apoptosis upon DNA damage (Hsieh et al., 2002; Pang et al., 2019). In humans, tRNA copy number variations can have phenotypic effects (Iben & Maraia, 2014; Kirchner & Ignatova, 2015). Since the two most differentiated SNPs in the sweep were located up‐ and downstream of a threonine tRNA, it is possible that they affect the transcription of the tRNA and therefore maybe affect phenotypic traits or physiological processes dependent on a certain amount of available threonine tRNA in the cell.

### 3.7 The selective sweep on Chr 13 contains a missense SNP in *cadm1*

Because of hitchhiking effects, where polymorphic loci in proximity on the chromosome are segregating together with the causative variant, it is often challenging to identify the specific variants that are under selection in a sweep region. However, nonsynonymous SNPs that alter the amino acid composition in functionally important protein domains or SNPs causing premature stop codons are therefore potential candidates for having significant phenotypic effects. Therefore, we divided the SNPs into functional categories and performed a screen for SNPs affecting the amino acid sequence of proteins. Screening for differentiated nonsense SNPs causing premature stop codons only revealed two such SNPs, in the genes *apoptotic protease‐activating factor 1* (*apaf1*, GeneID: 106576455, dAF = 0.55) on position Chr17:48969956 and *transmembrane protein 187* (*tmem187*, GeneID: 106609901, dAF = 0.57) on position Chr8:871592. A total of 112 missense SNPs were differentiated between the landlocked and anadromous populations (dAF > 50%), listed in File S2, covering 91 genes. Only 12 missense SNPs had dAF > 0.6, with the two most highly differentiated missense SNPs being located in the genes *cell adhesion molecule 1* (*cadm1*, File S2) and *collagen alpha‐2 type V* (*col5a2*, File S2).

**FIGURE 6:**
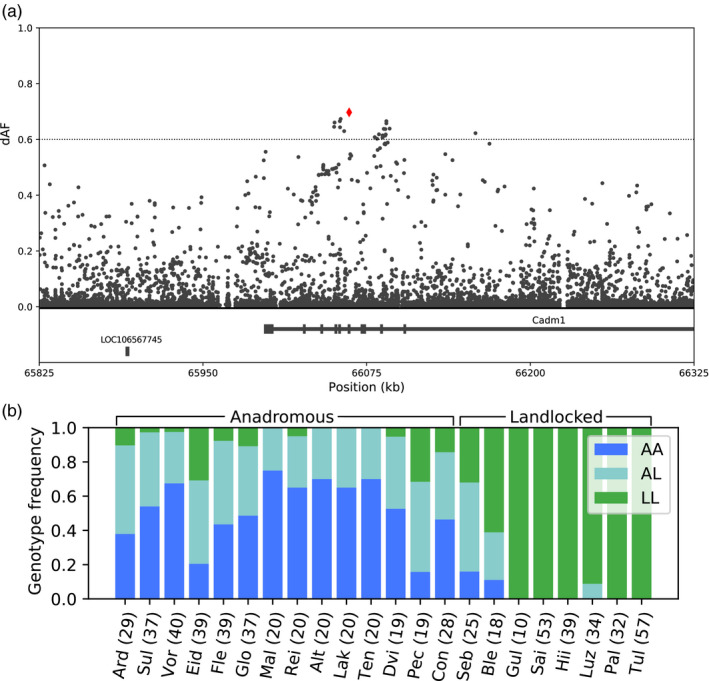
Detailed view of the genomic region on Chr 13 covering a missense SNP in *cadm1*. (a) Magnification showing 500 kb of the selective sweep on Chr 13. SNPs are shown as black dots, with the missense SNP (position 66,061,636) marked in red. The *x*‐axis shows the chromosomal positions given in kb and the *y*‐axis presents the difference in allele frequencies between the two groups (dAF). Genes from the reference annotation are shown in the bottom. (b) Genotype frequencies of the missense SNP in different populations based on genotyping of individual fish. AA = homozygous for the anadromous allele, LL = homozygous for the landlocked allele, AL = heterozygous. Population codes are explained in Table S1. The numbers in parentheses show the number of genotyped individuals from each population

A selective sweep on Chr 13 overlapped with *cadm1*, where the most differentiated SNP in the sweep, and the most differentiated missense SNP in the whole dataset, was changing the amino acid methionine in anadromous populations to a threonine in landlocked populations (Figure 6). The SNP is located in the second extracellular immunoglobulin domain, potentially affecting the structure and function of the protein. *cadm1* has been linked to several different functions, including behavior, neuron migration, immune system, and reproduction. In humans, missense mutations in the gene have been linked to autism (Zhiling et al., 2008), and mice lacking *cadm1* show impaired social interactions and increased anxiety (Takayanagi et al., 2010), in addition to male mice becoming sterile (Fujita et al., 2006). It also has a function in the immune system and has been reported in relation to human herpesvirus 8 (Hunte et al., 2018) and human T‐cell lymphotropic virus‐1 (Masuda et al., 2010; Pujari et al., 2015). Because Atlantic salmon *cadm1* is expressed in several tissues and highly expressed in the brain (Figure S4), it is difficult to speculate what function might be under selection, as behavior, immune response, and reproduction are all potentially relevant traits for adaptation to a life in different environments. Interestingly, it is known that landlocked salmon do not have the nerve innervation of important brain regions thought to be involved in downstream endocrine regulation of smolting (Stefansson et al., 2008). Most teleost fishes have a threonine in the position corresponding to the missense SNP, indicating that this may be the ancestral state; however, both amino acids can be found in different salmonids (File S4).

### 3.8 Selective sweep on Chr 5 is linked to ISA resistance

We also identified a selective sweep on Chr 5 (positions 8,550,000–8,800,000) which contains a SNP previously found to explain 5.83% of phenotype variation in resistance to infectious salmon anemia (ISA) in commercial Saint John River Atlantic salmon (Holborn et al., 2020). The sweep contains the two genes *sh3 domain‐containing ring finger 1* (*sh3rf1*) and *kinesin family member 3a* (*kif3a*). While *kif3a* is a microtubule motor protein involved in organelle organization and vesicle‐mediated transport, *sh3rf1* is assigned to the Reactome pathway (https://reactome.org) “Class I MHC mediated antigen processing & presentation” and can regulate T‐cell differentiation and activation in mice (Cunningham et al., 2013, 2016). Sh3rf1 has also been shown to be essential for production and release of HIV‐1 in humans (Alroy et al., 2005), suggesting a possible function in disease resistance in Atlantic salmon. Future studies will investigate which genetic variants in this sweep are associated with resistance to ISA.

The selective sweeps presented in this study provides a basis for identification of genetic variants with potential for increasing welfare of farmed animals. However, further studies are required to determine the precise function of genes and genetic variants under selection to be able to evaluate if any of these contribute to life‐history traits relevant for aquaculture, including growth, smoltification, and disease resistance. When selective sweeps have been connected to specific tissues, pathways, and traits in salmon, this knowledge can be further used to identify potential targets for introducing genetic variants possibly conferring relevant traits into farmed salmon strains to increase their robustness, for example by the use of marker‐assisted breeding or gene editing.

## 4 CONCLUSIONS

We describe genomic regions under divergent selection in anadromous and landlocked populations of Atlantic salmon across the Northern Hemisphere, and we report genes and genetic variants that may be of relevance for improving fish welfare in aquaculture production and for conservation and management related issues. The analyses were done using pooled whole‐genome sequencing of 12 anadromous and 6 landlocked salmon populations, which were used in a large genomewide association study. The study revealed 28 highly differentiated selective sweeps with SNPs close to fixation in all assayed landlocked populations, indicating parallel selection of alleles beneficial for a landlocked life cycle. Among the most interesting selective sweeps, we found *gpr132*, *cdca4*, *sertad2* and threonine tRNA in Chr 15, *cadm1* containing a highly differentiated missense SNP in Chr 13, and *ppargc1a* on Chr 18 which display increased expression in gills upon saltwater exposure. Further, we identified regions in the genome where the landlocked salmon show consistent signs of relaxed purifying selection, including the gene *igf1*, indicating genomic regions containing genes that are important during the seawater phase. Further studies will aim to characterize candidate genes and genotypes from the selective sweeps to pinpoint causative variants with potential for improving welfare in farmed salmon strains and to enhance our understanding of the underlying biology of transition into seawater.

## CONFLICT OF INTEREST

None declared.

## DISCLAIMERS

Any use of trade, firm, or product names is for descriptive purposes only and does not imply endorsement by the U.S. government.

## Supporting information

Supplementary Material

Supplementary Material

Supplementary Material

Supplementary Material

Supplementary Material

Supplementary Material

## Data Availability

All genomic sequence data used in this study have been deposited on SRA with BioProject ID PRJNA627844, with accession numbers for each sequenced pool listed in Table S1.

## References

[eva13129-bib-0001] Alroy, I. , Tuvia, S. , Greener, T. , Gordon, D. , Barr, H. M. , Taglicht, D. , Mandil‐Levin, R. , Ben‐Avraham, D. , Konforty, D. , Nir, A. , Levius, O. , Bicoviski, V. , Dori, M. , Cohen, S. , Yaar, L. , Erez, O. , Propheta‐Meiran, O. , Koskas, M. , Caspi‐Bachar, E. , & Reiss, Y. (2005). The trans‐Golgi network‐associated human ubiquitin‐protein ligase POSH is essential for HIV type 1 production. Proceedings of the National Academy of Sciences, 102(5), 1478–1483. 10.1073/pnas.0408717102 PMC545085 15659549

[eva13129-bib-0002] Altschul, S. F. , Madden, T. L. , Schaffer, A. A. , Zhang, J. , Zhang, Z. , Miller, W. , & Lipman, D. J. (1997). Gapped BLAST and PSI‐BLAST: A new generation of protein database search programs. Nucleic Acids Research, 25(17), 3389–3402. 10.1093/nar/25.17.3389 9254694 PMC146917

[eva13129-bib-0003] Ayllon, F. , Kjærner‐Semb, E. , Furmanek, T. , Wennevik, V. , Solberg, M. F. , Dahle, G. , Taranger, G. L. , Glover, K. A. , Almén, M. S. , Rubin, C. J. , Edvardsen, R. B. , & Wargelius, A. (2015). The vgll3 locus controls age at maturity in wild and domesticated Atlantic Salmon (*Salmo salar* L.) Males. PLoS Genetics, 11(11), e1005628 10.1371/journal.pgen.1005628 26551894 PMC4638356

[eva13129-bib-0004] Barson, N. J. , Aykanat, T. , Hindar, K. , Baranski, M. , Bolstad, G. H. , Fiske, P. , Jacq, C. , Jensen, A. J. , Johnston, S. E. , Karlsson, S. , Kent, M. , Moen, T. , Niemelä, E. , Nome, T. , Næsje, T. F. , Orell, P. , Romakkaniemi, A. , Sægrov, H. , Urdal, K. , & Primmer, C. R. (2015). Sex‐dependent dominance at a single locus maintains variation in age at maturity in salmon. Nature, 528(7582), 405–408. 10.1038/nature16062 26536110

[eva13129-bib-0005] Bjornsson, B. T. , Yamauchi, K. , Nishioka, R. S. , Deftos, L. J. , & Bern, H. A. (1987). Effects of hypophysectomy and subsequent hormonal replacement therapy on hormonal and osmoregulatory status of coho salmon, *Oncorhynchus* *Kisutch* . General and Comparative Endocrinology, 68(3), 421–430. 10.1016/0016-6480(87)90081-5 2830161

[eva13129-bib-0006] Bolick, D. T. , Skaflen, M. D. , Johnson, L. E. , Kwon, S.‐C. , Howatt, D. , Daugherty, A. , Ravichandran, K. S. , & Hedrick, C. C. (2009). G2A deficiency in mice promotes macrophage activation and atherosclerosis. Circulation Research, 104(3), 318–327. 10.1161/CIRCRESAHA.108.181131 19106413 PMC2716803

[eva13129-bib-0007] Bourret, V. , Kent, M. P. , Primmer, C. R. , Vasemagi, A. , Karlsson, S. , Hindar, K. , & Lien, S. (2013). SNP‐array reveals genome‐wide patterns of geographical and potential adaptive divergence across the natural range of Atlantic salmon (Salmo salar). Molecular Ecology, 22(3), 532–551. 10.1111/mec.12003 22967111

[eva13129-bib-0008] Carneiro, M. , Rubin, C.‐J. , Di Palma, F. , Albert, F. W. , Alfoldi, J. , Barrio, A. M. , Pielberg, G. , Rafati, N. , Sayyab, S. , Turner‐Maier, J. , Younis, S. , Afonso, S. , Aken, B. , Alves, J. M. , Barrell, D. , Bolet, G. , Boucher, S. , Burbano, H. A. , Campos, R. , & Andersson, L. (2014). Rabbit genome analysis reveals a polygenic basis for phenotypic change during domestication. Science, 345(6200), 1074–1079. 10.1126/science.1253714 25170157 PMC5421586

[eva13129-bib-0009] Chen, P. , Zuo, H. , Xiong, H. U. , Kolar, M. J. , Chu, Q. , Saghatelian, A. , Siegwart, D. J. , & Wan, Y. (2017). Gpr132 sensing of lactate mediates tumor‐macrophage interplay to promote breast cancer metastasis. Proceedings of the National Academy of Sciences, 114(3), 580–585. 10.1073/pnas.1614035114 PMC5255630 28049847

[eva13129-bib-0010] Chiang, C.‐Y. , Ching, Y.‐H. , Chang, T.‐Y. , Hu, L.‐S. , Yong, Y. S. , Keak, P. Y. , Mustika, I. , Lin, M.‐D. , & Liao, B.‐Y. (2020). Novel eye genes systematically discovered through an integrated analysis of mouse transcriptomes and phenome. Computational and Structural Biotechnology Journal, 18, 73–82. 10.1016/j.csbj.2019.12.009 31934309 PMC6951830

[eva13129-bib-0011] Cingolani, P. , Platts, A. , Wang, L. L. , Coon, M. , Nguyen, T. , Wang, L. , Land, S. J. , Lu, X. , & Ruden, D. M. (2012). A program for annotating and predicting the effects of single nucleotide polymorphisms, SnpEff: SNPs in the genome of Drosophila melanogaster strain w1118; iso‐2; iso‐3. Fly (Austin), 6(2), 80–92. 10.4161/fly.19695 22728672 PMC3679285

[eva13129-bib-0012] Cunningham, C. A. , Cardwell, L. N. , Guan, Y. , Teixeiro, E. , & Daniels, M. A. (2016). POSH regulates CD4+ T cell differentiation and survival. The Journal of Immunology, 196(10), 4003–4013. 10.4049/jimmunol.1501728 27084103 PMC4868786

[eva13129-bib-0013] Cunningham, C. A. , Knudson, K. M. , Peng, B. J. , Teixeiro, E. , & Daniels, M. A. (2013). The POSH/JIP‐1 scaffold network regulates TCR‐mediated JNK1 signals and effector function in CD8(+) T cells. European Journal of Immunology, 43(12), 3361–3371. 10.1002/eji.201343635 23963642

[eva13129-bib-0014] Dysvik, B. , & Jonassen, I. (2001). J‐Express: Exploring gene expression data using Java. Bioinformatics, 17(4), 369–370. 10.1093/bioinformatics/17.4.369 11301307

[eva13129-bib-0015] Felsenstein, J. (2005). Using the quantitative genetic threshold model for inferences between and within species. Philosophical Transactions of the Royal Society B‐Biological Sciences, 360(1459), 1427–1434. 10.1098/rstb.2005.1669 PMC1569509 16048785

[eva13129-bib-0016] Fernandez‐Marcos, P. J. , & Auwerx, J. (2011). Regulation of PGC‐1alpha, a nodal regulator of mitochondrial biogenesis. American Journal of Clinical Nutrition, 93(4), 884S–890. 10.3945/ajcn.110.001917 PMC3057551 21289221

[eva13129-bib-0017] Fujita, E. , Kouroku, Y. , Ozeki, S. , Tanabe, Y. , Toyama, Y. , Maekawa, M. , Kojima, N. , Senoo, H. , Toshimori, K. , & Momoi, T. (2006). Oligo‐astheno‐teratozoospermia in mice lacking RA175/TSLC1/SynCAM/IGSF4A, a cell adhesion molecule in the immunoglobulin superfamily. Molecular and Cellular Biology, 26(2), 718–726. 10.1128/MCB.26.2.718-726.2006 16382161 PMC1346906

[eva13129-bib-0018] Glover, K. A. , Solberg, M. F. , McGinnity, P. , Hindar, K. , Verspoor, E. , Coulson, M. W. , Hansen, M. M. , Araki, H. , Skaala, Ø. , & Svåsand, T. (2017). Half a century of genetic interaction between farmed and wild Atlantic salmon: Status of knowledge and unanswered questions. Fish and Fisheries, 18(5), 890–927. 10.1111/faf.12214

[eva13129-bib-0019] Hayashi, R. , Goto, Y. , Ikeda, R. , Yokoyama, K. K. , & Yoshida, K. (2006). CDCA4 is an E2F transcription factor family‐induced nuclear factor that regulates E2F‐dependent transcriptional activation and cell proliferation. Journal of Biological Chemistry, 281(47), 35633–35648. 10.1074/jbc.M603800200 16984923

[eva13129-bib-0020] Hoar, W. S. (2008). Fish Physiology (Vol. 11). Elsevier.

[eva13129-bib-0021] Holborn, M. K. , Ang, K. P. , Elliott, J. A. K. , Powell, F. , & Boulding, E. G. (2020). Genome wide analysis of infectious salmon anemia resistance in commercial Saint John River Atlantic salmon. Aquaculture, 514, 734514 10.1016/j.aquaculture.2019.734514

[eva13129-bib-0022] Hsieh, J.‐K. , Yap, D. , O’Connor, D. J. , Fogal, V. , Fallis, L. , Chan, F. , Zhong, S. , & Lu, X. (2002). Novel function of the cyclin A binding site of E2F in regulating p53‐induced apoptosis in response to DNA damage. Molecular and Cellular Biology, 22(1), 78–93. 10.1128/mcb.22.1.78-93.2002 11739724 PMC134205

[eva13129-bib-0023] Hunte, R. , Alonso, P. , Thomas, R. , Bazile, C. A. , Ramos, J. C. , van der Weyden, L. , & Shembade, N. (2018). CADM1 is essential for KSHV‐encoded vGPCR‐and vFLIP‐mediated chronic NF‐kappaB activation. PLoS Path, 14(4), e1006968 10.1371/journal.ppat.1006968 PMC5919438 29698475

[eva13129-bib-0024] Hutchings, J. A. , Ardren, W. R. , Barlaup, B. T. , Bergman, E. , Clarke, K. D. , Greenberg, L. A. , & Fraser, D. J. (2019). Life‐history variability and conservation status of landlocked Atlantic salmon: an overview. Canadian Journal of Fisheries and Aquatic Sciences, 76(10), 1697–1708.

[eva13129-bib-0025] Iben, J. R. , & Maraia, R. J. (2014). tRNA gene copy number variation in humans. Gene, 536(2), 376–384. 10.1016/j.gene.2013.11.049 24342656 PMC3941035

[eva13129-bib-0026] Iversen, M. , Mulugeta, T. , West, A. , Jørgensen, E. , Martin, S. A. M. , Sandve, S. R. , & Hazlerigg, D. (2020). Photoperiod‐dependent developmental reprogramming of the transcriptional response to seawater entry in Atlantic salmon (*Salmo salar*). bioRxiv, 2020.2003.2024.006510. 10.1101/2020.03.24.006510 PMC8049429 33710311

[eva13129-bib-0027] Kearse, M. , Moir, R. , Wilson, A. , Stones‐Havas, S. , Cheung, M. , Sturrock, S. , Buxton, S. , Cooper, A. , Markowitz, S. , Duran, C. , Thierer, T. , Ashton, B. , Meintjes, P. , & Drummond, A. (2012). Geneious Basic: An integrated and extendable desktop software platform for the organization and analysis of sequence data. Bioinformatics, 28(12), 1647–1649. 10.1093/bioinformatics/bts199 22543367 PMC3371832

[eva13129-bib-0028] Kirchner, S. , & Ignatova, Z. (2015). Emerging roles of tRNA in adaptive translation, signalling dynamics and disease. Nature Reviews Genetics, 16(2), 98–112. 10.1038/nrg3861 25534324

[eva13129-bib-0029] Kjærner‐Semb, E. , Ayllon, F. , Furmanek, T. , Wennevik, V. , Dahle, G. , Niemelä, E. , Ozerov, M. , Vähä, J.‐P. , Glover, K. A. , Rubin, C. J. , Wargelius, A. , & Edvardsen, R. B. (2016). Atlantic salmon populations reveal adaptive divergence of immune related genes ‐ a duplicated genome under selection. BMC Genomics, 17(1), 610 10.1186/s12864-016-2867-z 27515098 PMC4982270

[eva13129-bib-0030] Kofler, R. , Pandey, R. V. , & Schlotterer, C. (2011). PoPoolation2: Identifying differentiation between populations using sequencing of pooled DNA samples (Pool‐Seq). Bioinformatics, 27(24), 3435–3436. 10.1093/bioinformatics/btr589 22025480 PMC3232374

[eva13129-bib-0031] Langmead, B. , & Salzberg, S. L. (2012). Fast gapped‐read alignment with Bowtie 2. Nature Methods, 9(4), 357–359. 10.1038/nmeth.1923 22388286 PMC3322381

[eva13129-bib-0032] Leone, T. C. , Lehman, J. J. , Finck, B. N. , Schaeffer, P. J. , Wende, A. R. , Boudina, S. , & Kelly, D. P. (2005). PGC‐1alpha deficiency causes multi‐system energy metabolic derangements: Muscle dysfunction, abnormal weight control and hepatic steatosis. PLoS Biology, 3(4), e101 10.1371/journal.pbio.0030101 15760270 PMC1064854

[eva13129-bib-0033] Li, H. , Handsaker, B. , Wysoker, A. , Fennell, T. , Ruan, J. , Homer, N. , Marth, G. , Abecasis, G. , & Durbin, R. (2009). The sequence alignment/map format and SAMtools. Bioinformatics, 25(16), 2078–2079. 10.1093/bioinformatics/btp352 19505943 PMC2723002

[eva13129-bib-0034] Lien, S. , Koop, B. F. , Sandve, S. R. , Miller, J. R. , Kent, M. P. , Nome, T. , Hvidsten, T. R. , Leong, J. S. , Minkley, D. R. , Zimin, A. , Grammes, F. , Grove, H. , Gjuvsland, A. , Walenz, B. , Hermansen, R. A. , von Schalburg, K. , Rondeau, E. B. , Di Genova, A. , Samy, J. K. A. , & Davidson, W. S. (2016). The Atlantic salmon genome provides insights into rediploidization. Nature, 533(7602), 200–205. 10.1038/nature17164 27088604 PMC8127823

[eva13129-bib-0035] Liew, C. W. , Boucher, J. , Cheong, J. K. , Vernochet, C. , Koh, H.‐J. , Mallol, C. , Townsend, K. , Langin, D. , Kawamori, D. , Hu, J. , Tseng, Y.‐H. , Hellerstein, M. K. , Farmer, S. R. , Goodyear, L. , Doria, A. , Blüher, M. , Hsu, S.‐H. , & Kulkarni, R. N. (2013). Ablation of TRIP‐Br 2, a regulator of fat lipolysis, thermogenesis and oxidative metabolism, prevents diet‐induced obesity and insulin resistance. Nature Medicine, 19(2), 217–226. 10.1038/nm.3056 PMC3567215 23291629

[eva13129-bib-0036] Love, M. I. , Huber, W. , & Anders, S. (2014). Moderated estimation of fold change and dispersion for RNA‐seq data with DESeq2. Genome Biology, 15(12), 550 10.1186/s13059-014-0550-8 25516281 PMC4302049

[eva13129-bib-0037] Madsen, S. S. (1990). The role of cortisol and growth hormone in seawater adaptation and development of hypoosmoregulatory mechanisms in sea trout parr (*Salmo trutta trutta*). General and Comparative Endocrinology, 79(1), 1–11. 10.1016/0016-6480(90)90082-w 2162306

[eva13129-bib-0038] Maehr, T. , Wang, T. , Gonzalez Vecino, J. L. , Wadsworth, S. , & Secombes, C. J. (2012). Cloning and expression analysis of the transforming growth factor‐beta receptors type 1 and 2 in the rainbow trout Oncorhynchus mykiss. Developmental and Comparative Immunology, 37(1), 115–126. 10.1016/j.dci.2011.10.006 22057119

[eva13129-bib-0039] Marcel, M. (2011). Cutadapt removes adapter sequences from high‐throughput sequencing reads. Embnet Journal, 17, 10–12. 10.14806/ej.17.1.200

[eva13129-bib-0040] Masuda, M. , Maruyama, T. , Ohta, T. , Ito, A. , Hayashi, T. , Tsukasaki, K. , Kamihira, S. , Yamaoka, S. , Hoshino, H. , Yoshida, T. , Watanabe, T. , Stanbridge, E. J. , & Murakami, Y. (2010). CADM1 interacts with Tiam1 and promotes invasive phenotype of human T‐cell leukemia virus type I‐transformed cells and adult T‐cell leukemia cells. Journal of Biological Chemistry, 285(20), 15511–15522. 10.1074/jbc.M109.076653 PMC2865322 20215110

[eva13129-bib-0041] Maxime, V. , Boeuf, G. , Pennec, J. P. , & Peyraud, C. (1989). Comparative‐study of the energetic metabolism of Atlantic salmon (*Salmo‐Salar*) parr and smolts. Aquaculture, 82(1–4), 163–171. 10.1016/0044-8486(89)90405-5

[eva13129-bib-0042] McCormick, S. D. (1996). Effects of growth hormone and insulin‐like growth factor I on salinity tolerance and gill Na+, K+‐ATPase in Atlantic salmon (*Salmo salar*): Interaction with cortisol. General and Comparative Endocrinology, 101(1), 3–11. 10.1006/gcen.1996.0002 8713639

[eva13129-bib-0043] McCormick, S. D. (2001). Endocrine control of osmoregulation in teleost fish. American Zoologist, 41(4), 781–794. 10.1668/0003-1569(2001)041[0781:Ecooit]2.0.Co;2

[eva13129-bib-0044] McCormick, S. D. , Kelley, K. M. , Young, G. , Nishioka, R. S. , & Bern, H. A. (1992). Stimulation of coho salmon growth by insulin‐like growth factor I. General and Comparative Endocrinology, 86(3), 398–406. 10.1016/0016-6480(92)90064-q 1398004

[eva13129-bib-0045] McCormick, S. D. , Regish, A. M. , Ardren, W. R. , Bjornsson, B. T. , & Bernier, N. J. (2019). The evolutionary consequences for seawater performance and its hormonal control when anadromous Atlantic salmon become landlocked. Scientific Reports, 9(1), 968 10.1038/s41598-018-37608-1 30700821 PMC6353943

[eva13129-bib-0046] Nei, M. (1977). F‐statistics and analysis of gene diversity in subdivided populations. Annals of Human Genetics, 41(2), 225–233. 10.1111/j.1469-1809.1977.tb01918.x 596830

[eva13129-bib-0047] Nilsen, T. O. , Ebbesson, L. O. , Kiilerich, P. , Bjornsson, B. T. , Madsen, S. S. , McCormick, S. D. , & Stefansson, S. O. (2008). Endocrine systems in juvenile anadromous and landlocked Atlantic salmon (Salmo salar): Seasonal development and seawater acclimation. General and Comparative Endocrinology, 155(3), 762–772. 10.1016/j.ygcen.2007.08.006 17904138

[eva13129-bib-0048] Nilsen, T. O. , Ebbesson, L. O. , Madsen, S. S. , McCormick, S. D. , Andersson, E. , Bjornsson, B. T. , & Stefansson, S. O. (2007). Differential expression of gill Na+, K+‐ATPase alpha‐ and beta‐subunits, Na+, K+,2Cl‐ cotransporter and CFTR anion channel in juvenile anadromous and landlocked Atlantic salmon Salmo salar. Journal of Experimental Biology, 210(Pt 16), 2885–2896. 10.1242/jeb.002873 17690237

[eva13129-bib-0049] Pang, S. , Xu, Y. , Chen, J. , Li, G. , Huang, J. , & Wu, X. (2019). Knockdown of cell division cycle‐associated protein 4 expression inhibits proliferation of triple negative breast cancer MDA‐MB‐231 cells in vitro and in vivo. Oncology Letters, 17(5), 4393–4400. 10.3892/ol.2019.10077 30944632 PMC6444385

[eva13129-bib-0050] Pujari, R. , Hunte, R. , Thomas, R. , van der Weyden, L. , Rauch, D. , Ratner, L. , & Shembade, N. (2015). Human T‐cell leukemia virus type 1 (HTLV‐1) tax requires CADM1/TSLC1 for inactivation of the NF‐kappaB inhibitor A20 and constitutive NF‐kappaB signaling. PLoS Path, 11(3), e1004721 10.1371/journal.ppat.1004721 PMC4361615 25774694

[eva13129-bib-0051] Qanbari, S. , Strom, T. M. , Haberer, G. , Weigend, S. , Gheyas, A. A. , Turner, F. , Burt, D. W. , Preisinger, R. , Gianola, D. , & Simianer, H. (2012). A high resolution genome‐wide scan for significant selective sweeps: An application to pooled sequence data in laying chickens. PLoS One, 7(11), e49525 10.1371/journal.pone.0049525 23209582 PMC3510216

[eva13129-bib-0052] Quinlan, A. R. , & Hall, I. M. (2010). BEDTools: A flexible suite of utilities for comparing genomic features. Bioinformatics, 26(6), 841–842. 10.1093/bioinformatics/btq033 20110278 PMC2832824

[eva13129-bib-0053] Ronneseth, A. , Pettersen, E. F. , & Wergeland, H. I. (2005). Leucocytes of anadromous and landlocked strains of Atlantic salmon (*Salmo salar* L.) in the smolting period. Fish & Shellfish Immunology, 19(3), 229–239. 10.1016/j.fsi.2004.12.005 15820124

[eva13129-bib-0054] Rubin, C.‐J. , Zody, M. C. , Eriksson, J. , Meadows, J. R. S. , Sherwood, E. , Webster, M. T. , Jiang, L. , Ingman, M. , Sharpe, T. , Ka, S. , Hallböök, F. , Besnier, F. , Carlborg, Ö. , Bed’hom, B. , Tixier‐Boichard, M. , Jensen, P. , Siegel, P. , Lindblad‐Toh, K. , & Andersson, L. (2010). Whole‐genome resequencing reveals loci under selection during chicken domestication. Nature, 464(7288), 587–591. 10.1038/nature08832 20220755

[eva13129-bib-0055] Sakamoto, T. , McCormick, S. D. , & Hirano, T. (1993). Osmoregulatory actions of growth hormone and its mode of action in salmonids: A review. Fish Physiology and Biochemistry, 11(1–6), 155–164. 10.1007/BF00004562 24202472

[eva13129-bib-0056] Seidelin, M. , Madsen, S. S. , Byrialsen, A. , & Kristiansen, K. (1999). Effects of insulin‐like growth factor‐I and cortisol on Na+, K+‐ATPase expression in osmoregulatory tissues of brown trout (*Salmo trutta*). General and Comparative Endocrinology, 113(3), 331–342. 10.1006/gcen.1998.7225 10068495

[eva13129-bib-0057] Smith, J. M. , & Haigh, J. (1974). The hitch‐hiking effect of a favourable gene. Genetical Research, 23(1), 23–35. 10.1017/S0016672300014634 4407212

[eva13129-bib-0058] Sonawane, A. R. , Platig, J. , Fagny, M. , Chen, C.‐Y. , Paulson, J. N. , Lopes‐Ramos, C. M. , DeMeo, D. L. , Quackenbush, J. , Glass, K. , & Kuijjer, M. L. (2017). Understanding tissue‐specific gene regulation. Cell Reports, 21(4), 1077–1088. 10.1016/j.celrep.2017.10.001 29069589 PMC5828531

[eva13129-bib-0059] Stefansson, S. O. , Björnsson, B. T. , Ebbesson, L. O. E. , & McCormick, S. D. (2008). Smoltification In R. N. Finn , & B. G. Kapoor (Eds.), Fish Larval Physiology. Science Publishers.

[eva13129-bib-0060] Takayanagi, Y. , Fujita, E. , Yu, Z. , Yamagata, T. , Momoi, M. Y. , Momoi, T. , & Onaka, T. (2010). Impairment of social and emotional behaviors in Cadm1‐knockout mice. Biochemical and Biophysical Research Communications, 396(3), 703–708. 10.1016/j.bbrc.2010.04.165 20450890

[eva13129-bib-0061] Taranger, G. L. , Karlsen, Ø. , Bannister, R. J. , Glover, K. A. , Husa, V. , Karlsbakk, E. , Kvamme, B. O. , Boxaspen, K. K. , Bjørn, P. A. , Finstad, B. , Madhun, A. S. , Morton, H. C. , & Svåsand, T. (2015). Risk assessment of the environmental impact of Norwegian Atlantic salmon farming. ICES Journal of Marine Science, 72(3), 997–1021. 10.1093/icesjms/fsu132

[eva13129-bib-0062] Temple, S. E. , Veldhoen, K. M. , Phelan, J. T. , Veldhoen, N. J. , & Hawryshyn, C. W. (2008). Ontogenetic changes in photoreceptor opsin gene expression in coho salmon (*Oncorhynchus kisutch*, Walbaum). Journal of Experimental Biology, 211(Pt 24), 3879–3888. 10.1242/jeb.020289 19043060

[eva13129-bib-0063] Tonteri, A. , Titov, S. , Veselov, A. , Zubchenko, A. , Koskinen, M. T. , Lesbarreres, D. , & Primmer, C. R. (2005). Phylogeography of anadromous and non‐anadromous Atlantic salmon (*Salmo salar*) from northern Europe. Annales Zoologici Fennici, 42(1), 1–22.

[eva13129-bib-0064] Tsai, H. Y. , Hamilton, A. , Guy, D. R. , & Houston, R. D. (2014). Single nucleotide polymorphisms in the insulin‐like growth factor 1 (IGF1) gene are associated with growth‐related traits in farmed Atlantic salmon. Animal Genetics, 45(5), 709–715. 10.1111/age.12202 25090910 PMC4171758

[eva13129-bib-0065] Wennevik, V. , Quintela, M. , Skaala, O. , Verspoor, E. , Prusov, S. , & Glover, K. A. (2019). Population genetic analysis reveals a geographically limited transition zone between two genetically distinct Atlantic salmon lineages in Norway. Ecology and Evolution, 9(12), 6901–6921. 10.1002/ece3.5258 31380023 PMC6662299

[eva13129-bib-0066] Wood, A. W. , Duan, C. , & Bern, H. A. (2005). Insulin‐like growth factor signaling in fish. International Review of Cytology, 243, 215–285. 10.1016/S0074-7696(05)43004-1 15797461

[eva13129-bib-0067] Yano, A. , Nicol, B. , Jouanno, E. , Quillet, E. , Fostier, A. , Guyomard, R. , & Guiguen, Y. (2013). The sexually dimorphic on the Y‐chromosome gene (sdY) is a conserved male‐specific Y‐chromosome sequence in many salmonids. Evolutionary Applications, 6(3), 486–496. 23745140 10.1111/eva.12032 PMC3673476

[eva13129-bib-0068] Zhiling, Y. , Fujita, E. , Tanabe, Y. , Yamagata, T. , Momoi, T. , & Momoi, M. Y. (2008). Mutations in the gene encoding CADM1 are associated with autism spectrum disorder. Biochemical and Biophysical Research Communications, 377(3), 926–929. 10.1016/j.bbrc.2008.10.107 18957284

[eva13129-bib-0069] Zueva, K. J. , Lumme, J. , Veselov, A. E. , Kent, M. P. , & Primmer, C. R. (2018). Genomic signatures of parasite‐driven natural selection in north European Atlantic salmon (*Salmo salar*). Marine Genomics, 39, 26–38. 10.1016/j.margen.2018.01.001 29650372

