# Supplementary material for: Comparison of anadromous and landlocked Atlantic salmon genomes reveals signatures of parallel and relaxed selection across the Northern Hemisphere
Source: Evol Appl. 2020 Sep 23;14(2):446–61. doi: 10.1111/eva.13129 (PMC7896726; doi:10.1111/eva.13129)
Supplement: Supplementary file 1 — Supplementary Material [file EVA-14-446-s001.docx]

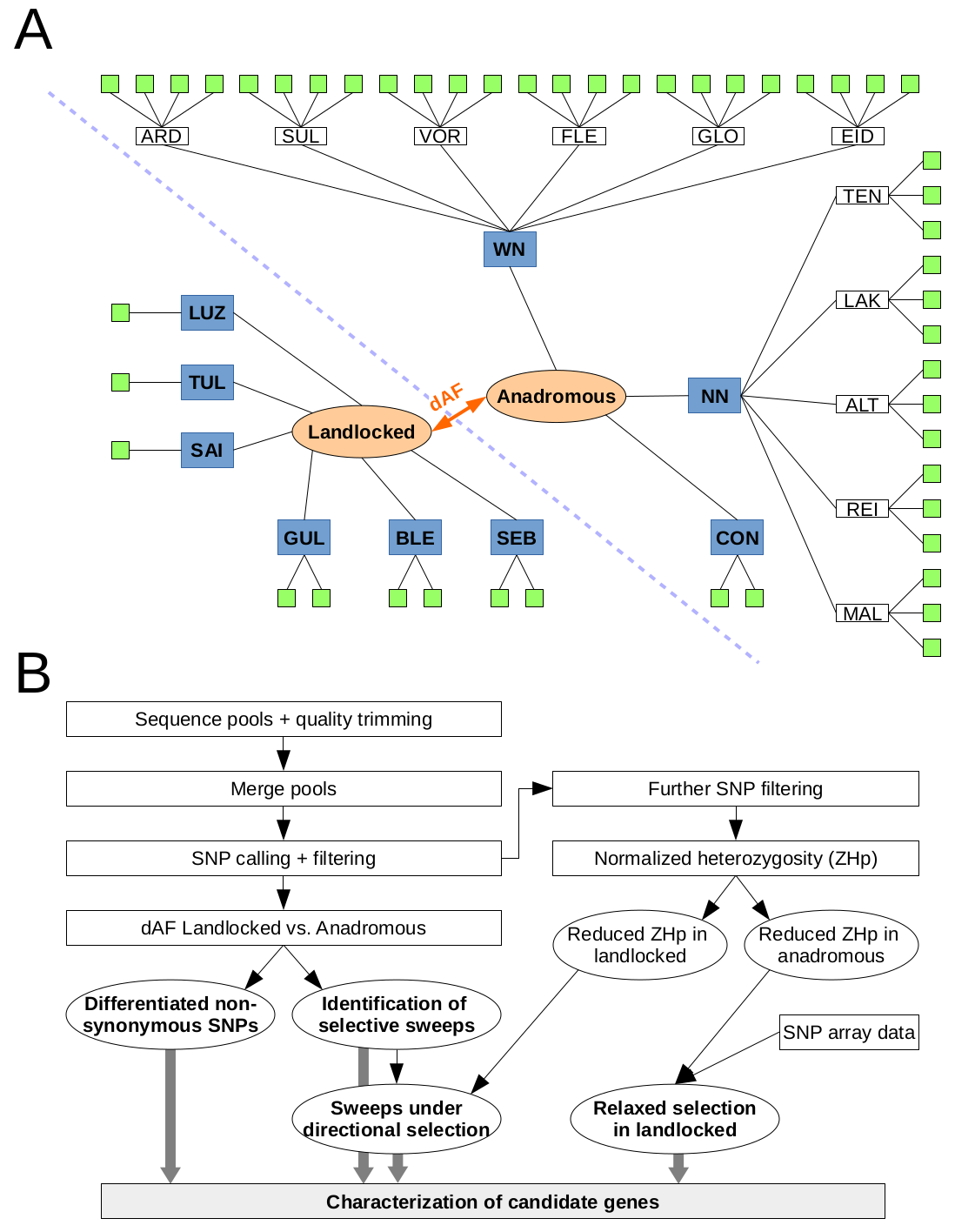


**Figure S1. Schematic overview of the study.** (**A**) Schematic diagram of how the data was organized on population level. Green boxes indicate pools of 10 individuals, white boxes indicate subpopulations, and blue boxes show the 9 populations included in the GWAS. Population codes are explained in Table S1. (**B**) Diagram presenting the structure of the analyses performed in this study. Boxes show analysis steps, ellipses show results, and bold font indicates major results.


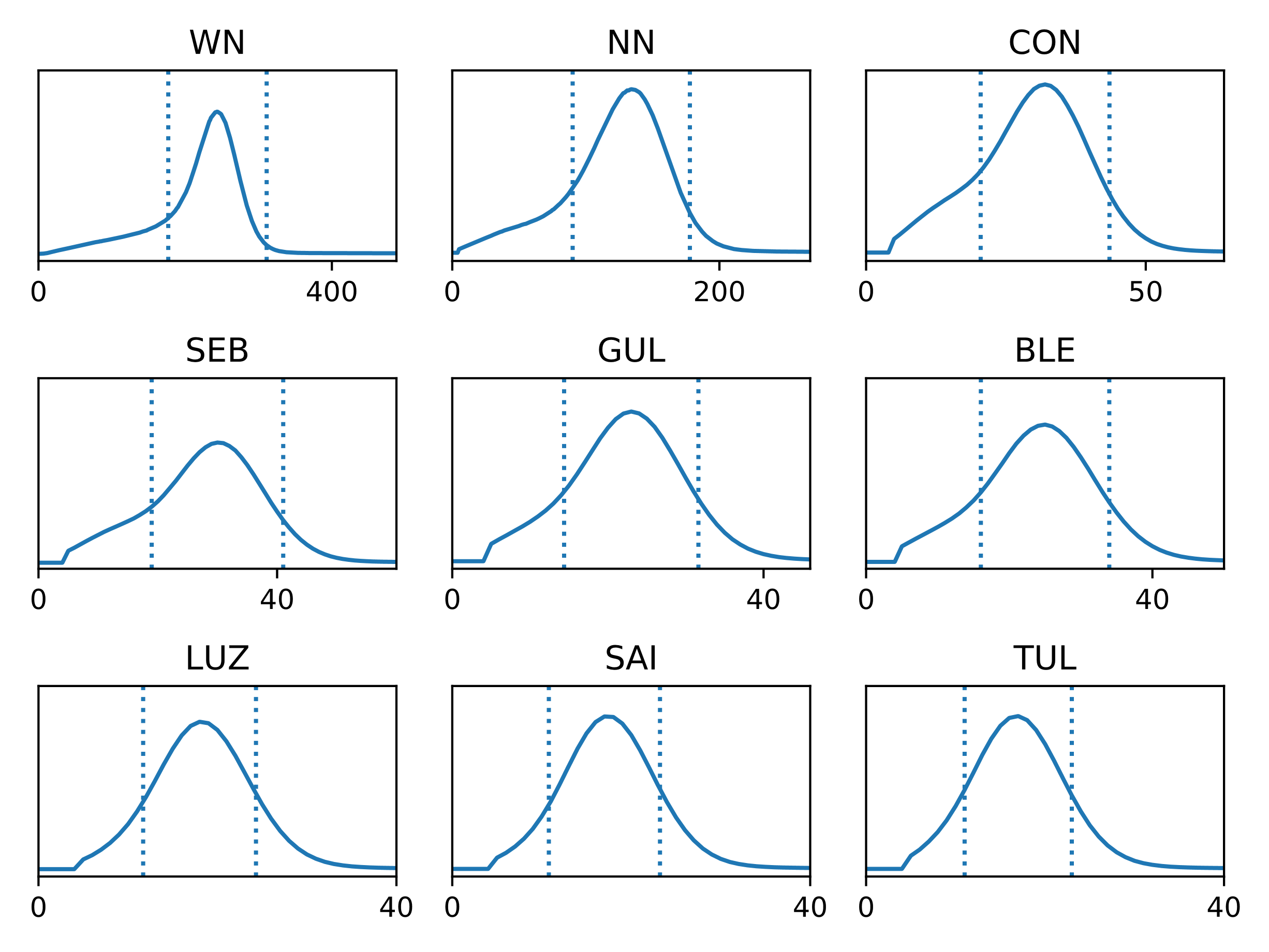
 **Figure S2. Depth of coverage profiles for SNPs.** Showing distributions of read depth for the SNPs (before the extra filtering step that was used for the ZHp analysis). The x-axis shows depth of coverage and the y-axis shows the proportion of SNPs having a given depth of coverage. The dotted lines indicate +/- 1 standard deviation from the peak. Values of the peaks for each population are listed in Table 1. Population codes are listed in Table 1 and Table S1.


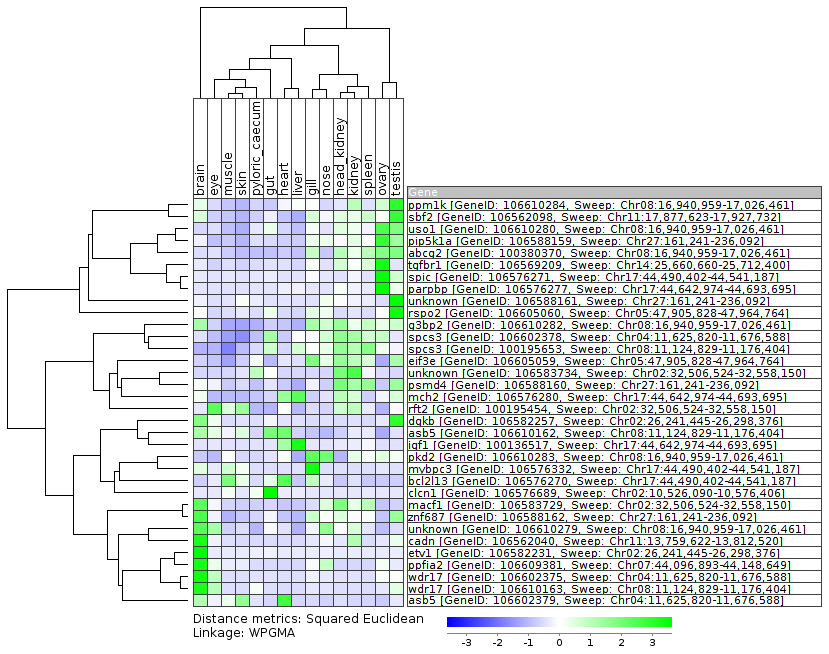
**Figure S3. Tissue distribution of gene expression for genes in regions with reduced heterozygosity in anadromous salmon.** Data is listed in Supporting File S2.


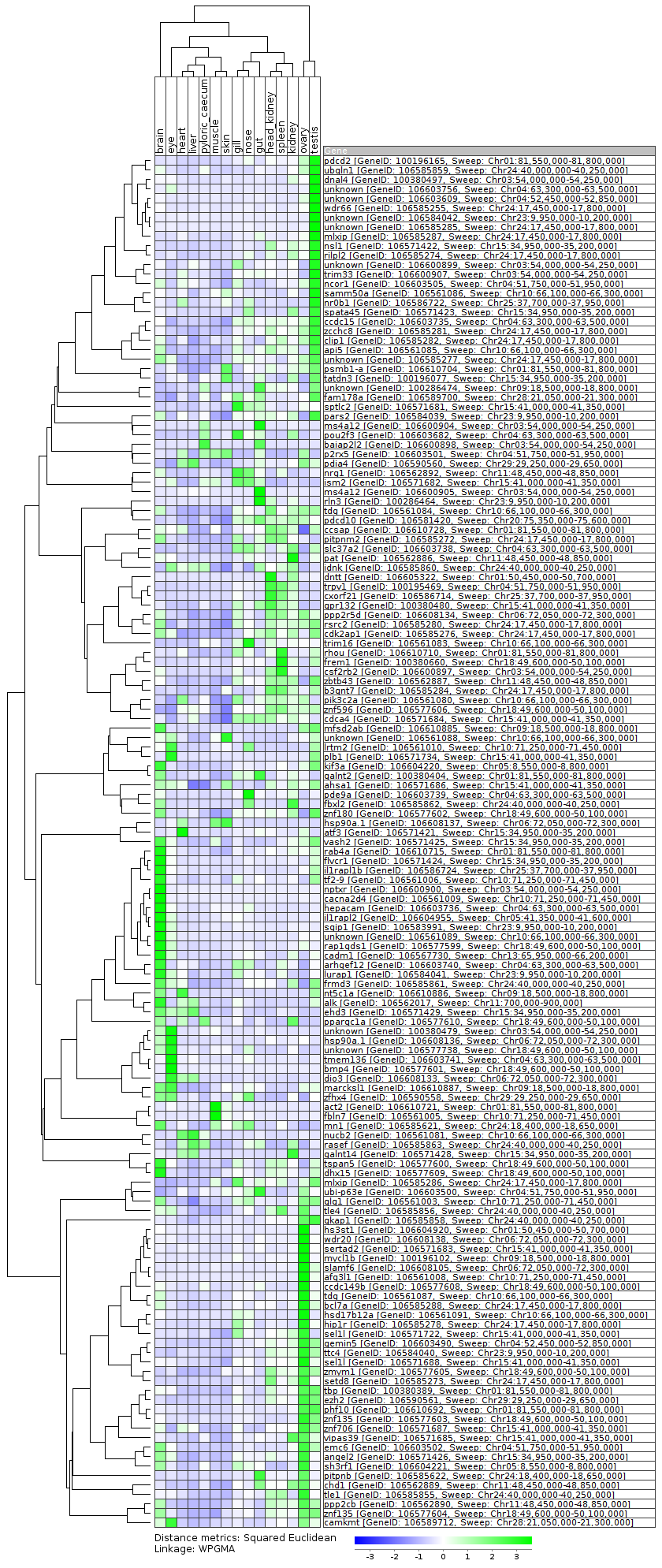


**Figure S4. Tissue distribution of gene expression for genes in 28 selective sweeps.** Data is listed in File S2.


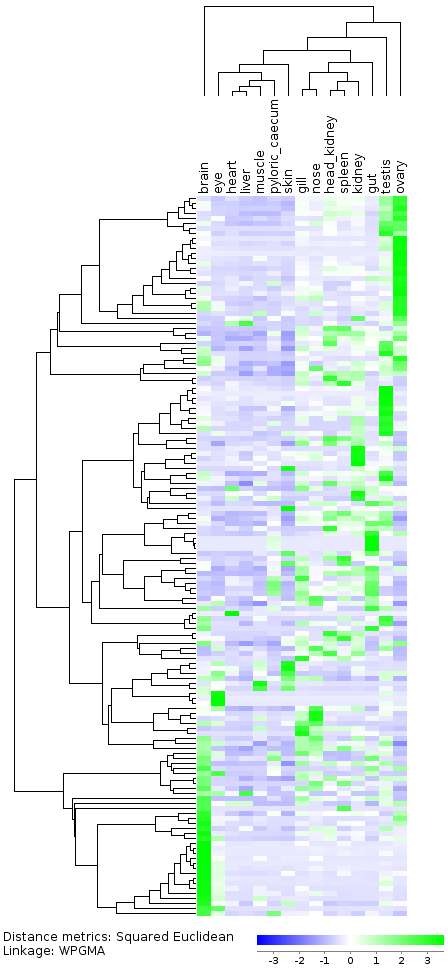


**Figure S5. Tissue distribution of gene expression for randomly selected genes.** Five genes from each chromosome were randomly chosen from the RNA-Seq dataset containing several different tissues. The distribution of gene expression is similar to that presented in Figure S4.

**
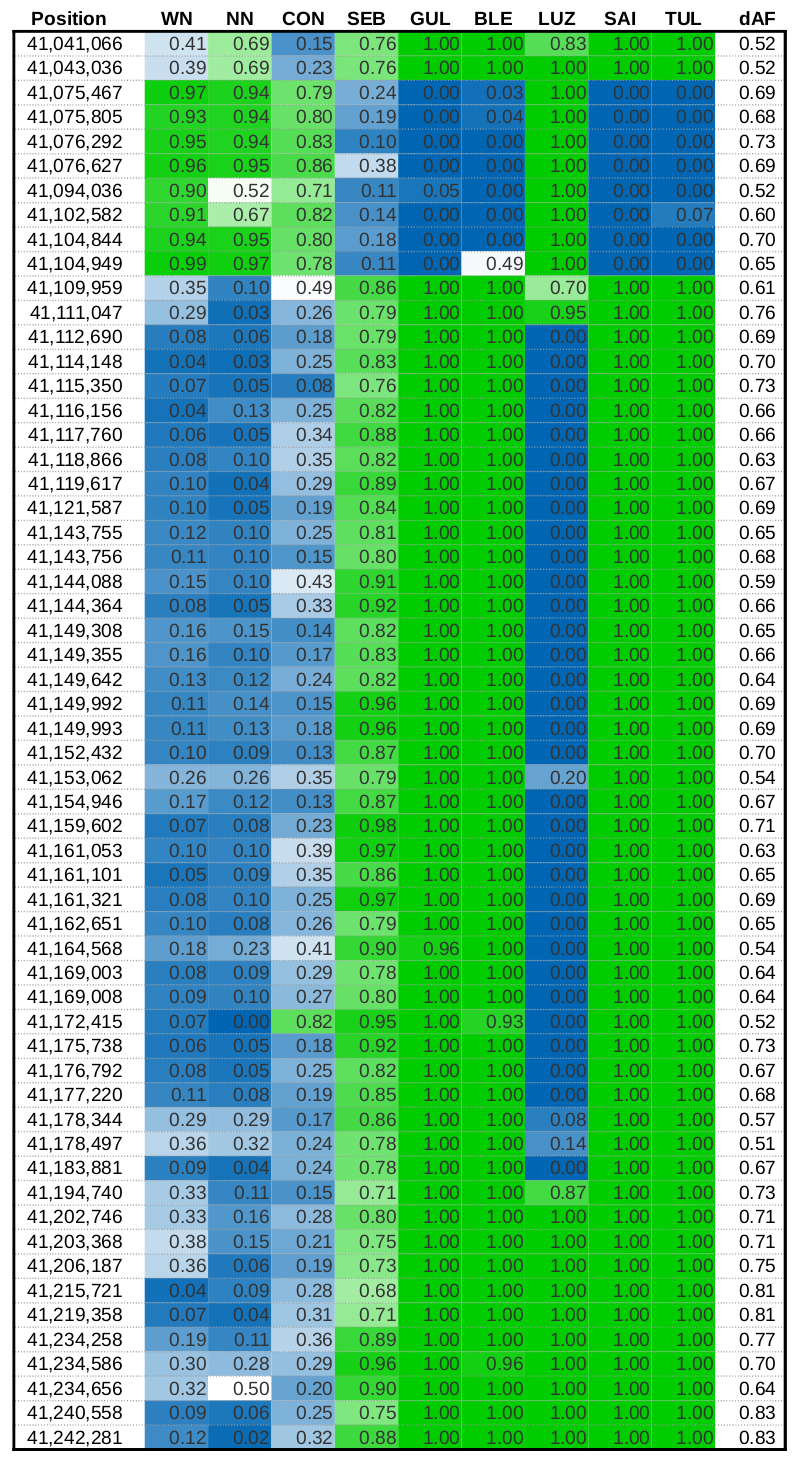

Figure S6. Allele frequencies in the selective sweep on Chr 15.** Showing reference allele frequencies of SNPs in the selective sweep on Chr 15 (positions 41,000,000 - 41,350,000) having dAF > 0.5 for the sequenced populations included in this study as a heatmap. Green and blue indicate SNPs with high and low reference allele frequencies, respectively. Differences of average allele frequencies (dAF) between landlocked and anadromous populations are shown in the last column on the right. Population codes are explained in Table 1 and Table S1.

**Table S1. Detailed overview of populations included in this study.**

| **Population code** | **Population name** | **Latitude, longitude** | **Anadromy** | **Group code** | **Group name** | **Sex** | **Country** | **Number of pools** | **Sequencing platform / read length** | **SRA BioSample accessions** |
| --- | --- | --- | --- | --- | --- | --- | --- | --- | --- | --- |
| ARD | Årdalselven | 59.144542, 6.170098 | Anadromous | WN | Western Norway | Male | Norway | 4 | Illumina HiSeq 2000 / 100 bp | SAMN03996247, SAMN03996246, SAMN03996245, SAMN03996244 |
| SUL | Suldalslågen | 59.481258, 6.251912 | Anadromous | WN | Western Norway | Male | Norway | 4 | Illumina HiSeq 2000 / 100 bp | SAMN03996565, SAMN03996564, SAMN03996563, SAMN03996562 |
| VOR | Vormo | 59.271843, 6.332463 | Anadromous | WN | Western Norway | Male | Norway | 4 | Illumina HiSeq 2000 / 100 bp | SAMN03996570, SAMN03996569, SAMN03996568, SAMN03996566 |
| FLE | Flekkeelven | 61.311164, 5.345375 | Anadromous | WN | Western Norway | Male | Norway | 4 | Illumina HiSeq 2000 / 100 bp | SAMN03996556, SAMN03996555, SAMN03996363, SAMN03996362 |
| GLO | Gloppenelven | 61.753802, 6.252684 | Anadromous | WN | Western Norway | Male | Norway | 4 | Illumina HiSeq 2000 / 100 bp | SAMN03996560, SAMN03996559, SAMN03996558, SAMN03996557 |
| EID | Eidselven | 61.901701, 5.984572 | Anadromous | WN | Western Norway | Male | Norway | 4 | Illumina HiSeq 2000 / 100 bp | SAMN03995935, SAMN03995934, SAMN03995933, SAMN03995901 |
| TEN | Tanaelven | 70.262086, 28.169989 | Anadromous | NN | Northern Norway | Male | Norway | 3 | Illumina HiSeq 2000 / 100 bp | SAMN05243777, SAMN05242666, SAMN04335018 |
| LAK | Lakselv | 70.078907, 24.958991 | Anadromous | NN | Northern Norway | Male | Norway | 3 | Illumina HiSeq 2000 / 100 bp | SAMN05243911, SAMN05243910, SAMN05243787 |
| ALT | Altaelva | 69.968016, 23.379053 | Anadromous | NN | Northern Norway | Male | Norway | 3 | Illumina HiSeq 2000 / 100 bp | SAMN05243914, SAMN05243913, SAMN05243912 |
| REI | Reisaelva | 69.779140, 21.014856 | Anadromous | NN | Northern Norway | Male | Norway | 3 | Illumina HiSeq 2000 / 100 bp | SAMN05243917, SAMN05243916, SAMN05243915 |
| MAL | Målselv | 69.200981, 18.485374 | Anadromous | NN | Northern Norway | Male | Norway | 3 | Illumina HiSeq 2000 / 100 bp | SAMN16057522, SAMN16057523, SAMN16057524 |
| CON | Connecticut River | 41.308874, -72.349323 | Anadromous | CON | Connecticut | Male + Female | USA | 2 | Illumina HiSeq X / 150 bp | SAMN16057525, SAMN16057526 |
| SEB | Sebago Lake | 43.852453, -70.561665 | Landlocked | SEB | Sebago Lake | Male + Female | USA | 2 | Illumina HiSeq X / 150 bp | SAMN16057527, SAMN16057528 |
| GUL | Gullspång | 58.993513, 14.099367 | Landlocked | GUL | Gullspång | Male + Female | Sweden | 2 | Illumina HiSeq 2500 / 125 bp | SAMN16057529, SAMN16057530 |
| BLE | Blege | 58.818953, 7.777799 | Landlocked | BLE | Blege | Male + Female | Norway | 2 | Illumina HiSeq 2500 / 125 bp | SAMN16057531, SAMN16057532 |
| SAI | Saimaa | 62.112838, 28.893957 | Landlocked | SAI | Saimaa | Mixed | Finland | 1 | Illumina HiSeq X / 150 bp | SAMN16057533 |
| TUL | Tulema | 61.356944, 31.841222 | Landlocked | TUL | Lake Ladoga | Mixed | Russia | 1 | Illumina HiSeq X / 150 bp | SAMN16057534 |
| LUZ | Luzhma | 63.220323, 33.285501 | Landlocked | LUZ | Luzhma | Mixed | Russia | 1 | Illumina HiSeq X / 150 bp | SAMN16057535 |
| DVI^†^ | Dvina | 64.102757, 41.726275 | Anadromous | DVI | Dvina | Male | Russia | - | - | - |
| PEC^†^ | Pechora | 67.687749, 52.540460 | Anadromous | PEC | Pechora | Male | Russia | - | - | - |
| HII^†^ | Hiitola | 61.199028, 29.770083 | Landlocked | HII | Lake Ladoga | Mixed | Russia | - | - | - |
| PAL^†^ | Pyalma | 62.404056, 35.873389 | Landlocked | PAL | Lake Onega | Mixed | Russia | - | - | - |

Note: Populations marked with ^†^ were only included for genotyping.

**Table S2. Primers and probes used for genotyping.** Custom TaqMan SNP Genotyping Assays (Thermo Fisher) used for genotyping, with probes targeting either the anadromous (A-allele) or the landlocked (L-allele) variants.

| **SNP** | **Primer/Probe type** | **Sequence (5’ - 3’)** |
| --- | --- | --- |
| Chr13:66061636 | Forward primer | GGCTGGTCACGGTGAACA |
| Chr13:66061636 | Reverse primer | CTGTCTTCATTTTCCTTCCCAGGTA |
| Chr13:66061636 | Vic probe (A-allele) | TGGTGTCGTACATCTCCT |
| Chr13:66061636 | Fam probe (L-allele) | TGGTGTCGTACGTCTCCT |
| Chr15:41215721 | Forward primer | ACTCAGTCCTGAAAAGAAGCCATTC |
| Chr15:41215721 | Reverse primer | GCAGTGTCAGTCCAGTCTTAGG |
| Chr15:41215721 | Vic probe (L-allele) | CACATCTGGATTTAAC |
| Chr15:41215721 | Fam probe (A-allele) | ACATCTGCATTTAAC |

**Table S3. Regions with reduced heterozygosity in landlocked salmon.** Listing regions showing consistently reduced heterozygosity in landlocked compared to anadromous populations (intersect of our data and the data from Zueva et al., 2018 (Zueva et al., 2018)). Gene symbols of genes from the reference annotation are shown as obtained from the annotation against Swiss-Prot, where genes lacking a gene symbol are indicated by “unknown”. A detailed description of the genes can be found in File S2.

| **Chromosome** | **Chromosomal region** | **Region length (bp)** | **Genes** |
| --- | --- | --- | --- |
| 1 | 37,639,629 – 37,726,238 | 86 609 |  |
| 1 | 39,760,768 – 39,815,777 | 55 009 | Mta1, tmem229b |
| 1 | 48,350,297 – 48,438,962 | 88 665 | unknown, Sirt1, fam83h |
| 1 | 57,915,735 – 57,969,168 | 53 433 | CAMK2G |
| 1 | 60,225,931 – 60,284,906 | 58 975 | PCSK2 |
| 1 | 117,930,739 – 117,980,817 | 50 078 | AMACR |
| 1 | 152,486,602 – 152,544,400 | 57 798 | LMX1B |
| 2 | 17,353,744 – 17,408,755 | 55 011 | unknown, NOD1, znrf2 |
| 2 | 24,755,780 – 24,809,640 | 53 860 | Sv2a |
| 3 | 14,379,901 – 14,430,139 | 50 238 | PGBD4, PGBD4, dvl3 |
| 3 | 15,995,251 – 16,060,483 | 65 232 | PGBD4, Eps15, Calr, MRPL37, Acot11, SAP130 |
| 3 | 23,013,876 – 23,067,147 | 53 271 | Klf2 |
| 3 | 34,431,370 – 34,484,484 | 53 114 | Ipo13, IPO13 |
| 4 | 49,614,173 – 49,670,882 | 56 709 | cxadr |
| 4 | 60,000,995 – 60,094,100 | 93 105 | OPCML |
| 6 | 50,731,777 – 50,786,848 | 55 071 | E2F6, ASAP2, mboat2 |
| 6 | 62,029,961 – 62,083,296 | 53 335 | disp1, unknown |
| 6 | 68,599,010 – 68,675,653 | 76 643 | FKBP1B, si:dkeyp-115a10.2, mfsd2b, UBXN2A, ATAD2B |
| 7 | 19,435,468 – 19,491,064 | 55 596 | ENDOD1, ENDOD1, Gvin1, Prf1, ENDOD1 |
| 7 | 23,868,532 – 23,919,650 | 51 118 | Cbln1, Khnyn |
| 7 | 42,681,343 – 42,732,236 | 50 893 | ascl1a, unknown |
| 9 | 24,785,821 – 24,906,711 | 120 890 | unknown, DHRS7, pcnxl4, Ppm1a, SIX6 |
| 9 | 27,282,675 – 27,344,690 | 62 015 | sobpa, Pdss2, ROCK2 |
| 9 | 60,596,024 – 60,660,815 | 64 791 | CRYBB1, unknown, ZNF146, Smtnl2, Ctnnd1 |
| 9 | 62,318,802 – 62,399,594 | 80 792 |  |
| 9 | 104,928,556 – 104,981,887 | 53 331 | apoa1, Cep164, Bace1 |
| 10 | 29,914,149 – 29,964,206 | 50 057 | PRDM5 |
| 10 | 77,994,914 – 78,063,739 | 68 825 | CDH13 |
| 11 | 6,311,055 – 6,364,674 | 53 619 | slc39a1, Rlbp1 |
| 11 | 13,288,223 – 13,347,215 | 58 992 | TSHZ3, unknown |
| 11 | 67,037,328 – 67,093,046 | 55 718 | ASTN2 |
| 12 | 75,756,868 – 75,808,978 | 52 110 |  |
| 13 | 68,080,852 – 68,146,208 | 65 356 | NSUN5, TBL2, YPEL1, POM121C, bcl7ba, Crybg3 |
| 13 | 82,890,564 – 82,947,591 | 57 027 | KSR1, CUEDC1 |
| 14 | 32,602,921 – 32,653,180 | 50 259 | Mmp16 |
| 14 | 60,799,690 – 60,852,978 | 53 288 | psmb4, RFX5, pi4kb |
| 14 | 86,017,756 – 86,069,531 | 51 775 | Srsf4, TMEM200B |
| 15 | 24,110,737 – 24,163,679 | 52 942 | RSPH3, EZR, Tagap |
| 15 | 93,264,531 – 93,323,719 | 59 188 | krt18, krt18 |
| 15 | 97,127,315 – 97,178,558 | 51 243 | NGRN, Nfya, sort1, Ahcyl1 |
| 16 | 32,064,947 – 32,128,430 | 63 483 | unknown, znf423 |
| 17 | 36,411,539 – 36,466,771 | 55 232 | TFEC, AKR1B1 |
| 18 | 12,363,530 – 12,440,243 | 76 713 | unknown, MMRN2, Bmpr1a |
| 18 | 12,557,672 – 12,614,408 | 56 736 | unknown, opn4a |
| 18 | 12,714,703 – 12,765,096 | 50 393 | USP54 |
| 18 | 23,731,855 – 23,785,327 | 53 472 | GFRA1, Atrnl1 |
| 18 | 41,578,844 – 41,629,761 | 50 917 |  |
| 20 | 8,360,387 – 8,410,630 | 50 243 | unknown, fam172a |
| 21 | 6,846,457 – 6,920,509 | 74 052 | Obscn |
| 21 | 26,126,684 – 26,199,282 | 72 598 | ATP6AP2, IMPG2, Senp7, BCOR |
| 22 | 9,402,193 – 9,454,004 | 51 811 | RHOC, FAM19A2, PPM1H |
| 23 | 3,685,085 – 3,742,994 | 57 909 | Wwtr1 |
| 23 | 9,269,999 – 9,326,523 | 56 524 | Raver2 |
| 23 | 27,394,207 – 27,445,842 | 51 635 | fnbp1l, BCAR3 |
| 24 | 9,250,927 – 9,327,691 | 76 764 | Tnfrsf22, UNC5D, loxl2b |
| 24 | 13,937,065 – 13,987,655 | 50 590 | ENKD1, ctp, RPL6, Acaa2, Mrpl40, unknown, polb, UFD1L |
| 24 | 17,681,277 – 17,739,979 | 58 702 | unknown, B3gnt7, MLXIP, CLIP1 |
| 25 | 38,122,335 – 38,186,930 | 64 595 | SPRYD7, ARL11, Phf6, Kpna3 |
| 27 | 18,597,255 – 18,650,223 | 52 968 | STMN1, PKIA |
| 29 | 11,985,909 – 12,063,506 | 77 597 | Tyms, colec12, CLUL1, yes1 |
| 29 | 16,194,979 – 16,245,405 | 50 426 | THNSL1, PRTFDC1, ARHGAP21 |
| 29 | 20,325,167 – 20,376,341 | 51 174 | EEF1E1 |
| 29 | 28,029,912 – 28,081,456 | 51 544 |  |

**Supporting File S1. Regions with reduced heterozygosity in the sequencing data.** Excel file containing lists of regions with reduced ZHp in anadromous and landlocked salmon identified from sequencing data used in this study. The file also lists genomic regions showing reduced ZHp in anadromous and landlocked salmon in the dataset from Zueva et al. 2018 ([Zueva et al., 2018](#_ENREF_67)).

**Supporting File S2. Detailed information on genes in identified regions.** Excel file containing details on genes in genomic regions of interest that were discovered in this study. The file includes gene names, GeneIDs, locations, annotations, and normalized read counts for tissue distributions. Genes in selective sweeps, genes with missense SNPs (including dAF values) and genes in regions with reduced ZHp in anadromous and landlocked salmon that overlapped between our data and the data from Zueva et al., 2018 are shown in separate tabs.

**Supporting File S3. Expression of candidate genes in gills after saltwater exposure.** Excel file containing RNA-Seq gene expression levels of genes in genomic regions of interest from salmon subjected to 24h saltwater (SW) exposure and salmon kept in freshwater (FW). The file includes gene names, GeneIDs, locations, annotations, normalized read counts, and statistical significance for contrasts between FW and SW at each sampling point. Genes in selective sweeps, genes with missense SNPs (including dAF values) and genes in regions with reduced ZHp in anadromous salmon are shown in separate tabs.

**Supporting File S4. Ancestral state of *cadm1* missense SNP.** Excel file listing of amino acids at positions corresponding to the missense SNP in *cadm1* in other teleost fishes. Amino acids from various reference sequences from salmonids and teleost fishes (without salmonids) are shown in separate tabs.
